# A multi-strategy improved rime optimization algorithm for three-dimensional USV path planning and global optimization

**DOI:** 10.1038/s41598-024-63188-4

**Published:** 2024-06-01

**Authors:** Gaoquan Gu, Jingjun Lou, Haibo Wan

**Affiliations:** College of Naval Architecture and Ocean Engineering, Naval University of Engineering, Wuhan, 430033 Hubei China

**Keywords:** Rime optimization algorithm, Metaheuristic algorithm, Numerical optimization, Three-dimensional USV path planning, Engineering, Mechanical engineering, Mathematics and computing, Computer science, Statistics

## Abstract

The RIME optimization algorithm (RIME) represents an advanced optimization technique. However, it suffers from issues such as slow convergence speed and susceptibility to falling into local optima. In response to these shortcomings, we propose a multi-strategy enhanced version known as the multi-strategy improved RIME optimization algorithm (MIRIME). Firstly, the Tent chaotic map is utilized to initialize the population, laying the groundwork for global optimization. Secondly, we introduce an adaptive update strategy based on leadership and the dynamic centroid, facilitating the swarm's exploitation in a more favorable direction. To address the problem of population scarcity in later iterations, the lens imaging opposition-based learning control strategy is introduced to enhance population diversity and ensure convergence accuracy. The proposed centroid boundary control strategy not only limits the search boundaries of individuals but also effectively enhances the algorithm's search focus and efficiency. Finally, to demonstrate the performance of MIRIME, we employ CEC 2017 and CEC 2022 test suites to compare it with 11 popular algorithms across different dimensions, verifying its effectiveness. Additionally, to assess the method's practical feasibility, we apply MIRIME to solve the three-dimensional path planning problem for unmanned surface vehicles. Experimental results indicate that MIRIME outperforms other competing algorithms in terms of solution quality and stability, highlighting its superior application potential.

## Introduction

Roughly two-thirds of the Earth’s surface is covered by oceans. Unmanned Surface Vehicle (USV) serves as pivotal instruments for oceanic exploration and monitoring, with their trajectory planning being imperative for ensuring efficient and safe maritime operations^1,2^. Path planning encompasses not only the selection of optimal routes but also the meticulous consideration of various offshore environmental complexities, including obstacles, ocean currents, wind speed, and other factors. Such considerations impose stringent requirements on the navigation accuracy and safety protocols of unmanned vessels^3^. Metaheuristic algorithms have emerged as a prominent research focus within the realm of unmanned vessel path planning, owing to their efficacy and adaptability in tackling such intricate optimization dilemmas^4,5^.

Metaheuristics are commonly classified into four categories^6^: physical heuristics, swarm intelligence algorithms, evolutionary algorithms, and human behavior heuristics. Among these, physical heuristic algorithms draw inspiration from physical laws and chemical reaction mechanisms. For instance, the simulated annealing algorithm, pioneered by Kirkpatrick et al.^7^ , is rooted in the concept of substances attaining thermal equilibrium. Swarm intelligence algorithms emulate the collective behaviors observed in social creatures in nature^8^ , such as the particle swarm optimization algorithm proposed by Eberhart et al.^9^ , which is based on the flocking behavior of birds. Evolutionary algorithms leverage selection and genetic mechanisms found in nature^10^. For example, the genetic algorithm, conceptualized by Holland^11^, is inspired by Darwin's theory of natural selection. Lastly, human behavior heuristics, like the teaching optimization algorithm developed by Rao et al.^12^, mimic the interactive processes observed in classrooms. Despite the variance in heuristic sources and definitions, these algorithms share a fundamental trait^13,14^: decomposing search into two key phases: exploration and exploitation. During the exploration stage, an extensive search of the solution space is conducted to identify all potentially promising regions. Subsequently, the exploitation phase delves deeper into these identified regions, aiming to uncover the optimal solution.

In recent years, metaheuristic algorithms have developed rapidly. For instance, Parizi et al.^15^ simulated the mating behavior of woodpeckers and proposed the Woodpecker Mating Algorithm (WMA). Inspired by the Lichtenberg pattern in the process of lightning discharge, Pereira et al.^16^ developed a new global optimization algorithm called Lichtenberg Algorithm (LA). Abdel-Basset et al.^17^, drawing inspiration from the light wave properties elucidated by Young's double-slit experiments, introduced the Young's Double Slit Experimental Optimizer (YDSE), effectively balancing exploration and exploitation in solving complex optimization problems. Deng et al.^18^ proposed the Snow Ablation Optimizer (SAO) for numerical optimization and engineering design, primarily mimicking the sublimation and melting behavior of snow. Abouhawwash et al.^19^ introduced the Kepler Optimization Algorithm (KOA), a novel physically-based algorithm inspired by Kepler's laws of planetary motion, used for predicting the position and velocity of planets at any given time. Jia et al.^20^ devised the Crayfish Optimization Algorithm (COA), simulating crayfish behaviors such as escape, competition, and foraging during summer. Inspired by the magnification ability of the optical microscope to the target object, Cheng et al.^21^ developed the Optical Microscope Algorithm (OMA), inspired by the magnification ability of optical microscopes on target objects. Han et al.^22^ proposed the Walrus Optimizer (WO), inspired by the key signals (danger and safety signals) received by walruses, influencing their migration, breeding, habitat, foraging, aggregation, and escape behaviors. The Newton–Raphson-Based Optimizer (NRBO) proposed by Sowmya et al.^23^ is derived from the Newton–Raphson-based search rule (NRSR) and the Trap Avoidance Operator (TAO). These algorithms, along with several other techniques, have found successful applications in unmanned ships and various other fields^24–27^.

The three-dimensional path planning of unmanned vessels is classified as an NP-Hard problem, for which metaheuristic algorithms serve as effective tools in finding approximate global optimal solutions. These algorithms yield satisfactory results within limited timeframes, rendering them highly suitable for tackling complex problems. Particularly, they demonstrate significant advantages in addressing the intricate three-dimensional path planning issues encountered by large-scale unmanned ships. Numerous researchers have leveraged these algorithms to optimize the path planning of unmanned vessels, thereby achieving notable success in overcoming these complex challenges^28,29^. Nonetheless, it is imperative to acknowledge the existence of the No Free Lunch theorem (NFL)^30^, underscoring the importance of developing specialized algorithms tailored to the unique properties of the problem to attain optimal and efficient results. For example, Liu et al.^31^ proposed an enhanced Aquila optimizer (TEOA) based on tent chaotic maps and new rules. Jia et al.^32^ proposed an improved Sand Cat Swarm Optimization (MSCSO) algorithm.

The Rime optimization algorithm (RIME), proposed by Su et al.^33^ in 2023, is a physics-based approach. It achieves exploration and exploitation behaviors in optimization by simulating the soft and hard frost growth processes of rime, thus constructing the soft-rime search strategy and the hard-rime penetration mechanism. Additionally, enhancements have been made to the greedy selection mechanism within the algorithm, and population updates during the optimal solution selection stage aim to improve RIME's exploit performance. Due to the excellent performance of the RIME, Yousri et al.^34^ used RIME optimizer to optimize the layout of thermoelectric generator array reconstruction to reduce the overall power consumption and maximize the generated power. Salman et al.^35^ used RIME algorithm to adjust the controller parameters. Realize the automatic control of complex hybrid power system. Nevertheless, the optimizer occasionally suffers from slow convergence speed, susceptibility to stalling in local optima, and inadequate handling of practical problems. For example, Zhang et al.^36^ proposed a RIME algorithm based on vertical and horizontal cross search strategy (CCRIME) to improve the quality of solutions obtained by RIME algorithm and further enhance its search ability. Zhong et al.^37^ proposed a strengthened RIME (SRIME) to solve the problem that RIME algorithm is easy to fall into local optimum, which leads to the stagnation of optimization. Inspired by the above research, we are motivated to develop a multi-strategy improved Rime optimizer (MIRIME). The main contributions of this paper are as follows:

1. In the initial population stage, the Tent chaotic map was introduced to render the distribution of individuals within the search space more rational, thereby enhancing the algorithm's ability to locate the optimal value.
2. A novel update rule, combining the leader and the centroid, is proposed to guide individuals towards the optimal solution and expedite the convergence speed of the algorithm.
3. The lens opposition-based learning strategy is introduced to improve the convergence speed while ensuring accuracy.
4. A novel centroid boundary adjustment strategy is proposed, effectively leveraging information from the entire population and reducing the risk of the algorithm converging to local optima.
5. MIRIME is evaluated on the CEC2017 and CEC2022 test suites with different dimensions.
6. MIRIME is applied to the 3D path planning of unmanned vessels, and the results demonstrate the effectiveness and robustness of MIRIME in solving real-world problems.

The remainder of this paper is structured as follows: Section "RIME optimizer" reviews the original RIME algorithm. Section "The proposed MIRIME" provides a detailed exposition of the proposed MIRIME. In Section "Analysis of experimental results", numerical experiments and analysis are conducted. Section "MIRIME algorithm for 3D path planning of unmanned vessel" presents the 3D path planning model and simulation analysis of unmanned vessels. Finally, Section "Summary and outlook" offers a summary and outlook.

## RIME optimizer

This section briefly outlines the inspiration and mathematical model of RIME.

### Inspiration for RIME

**Figure 1:**
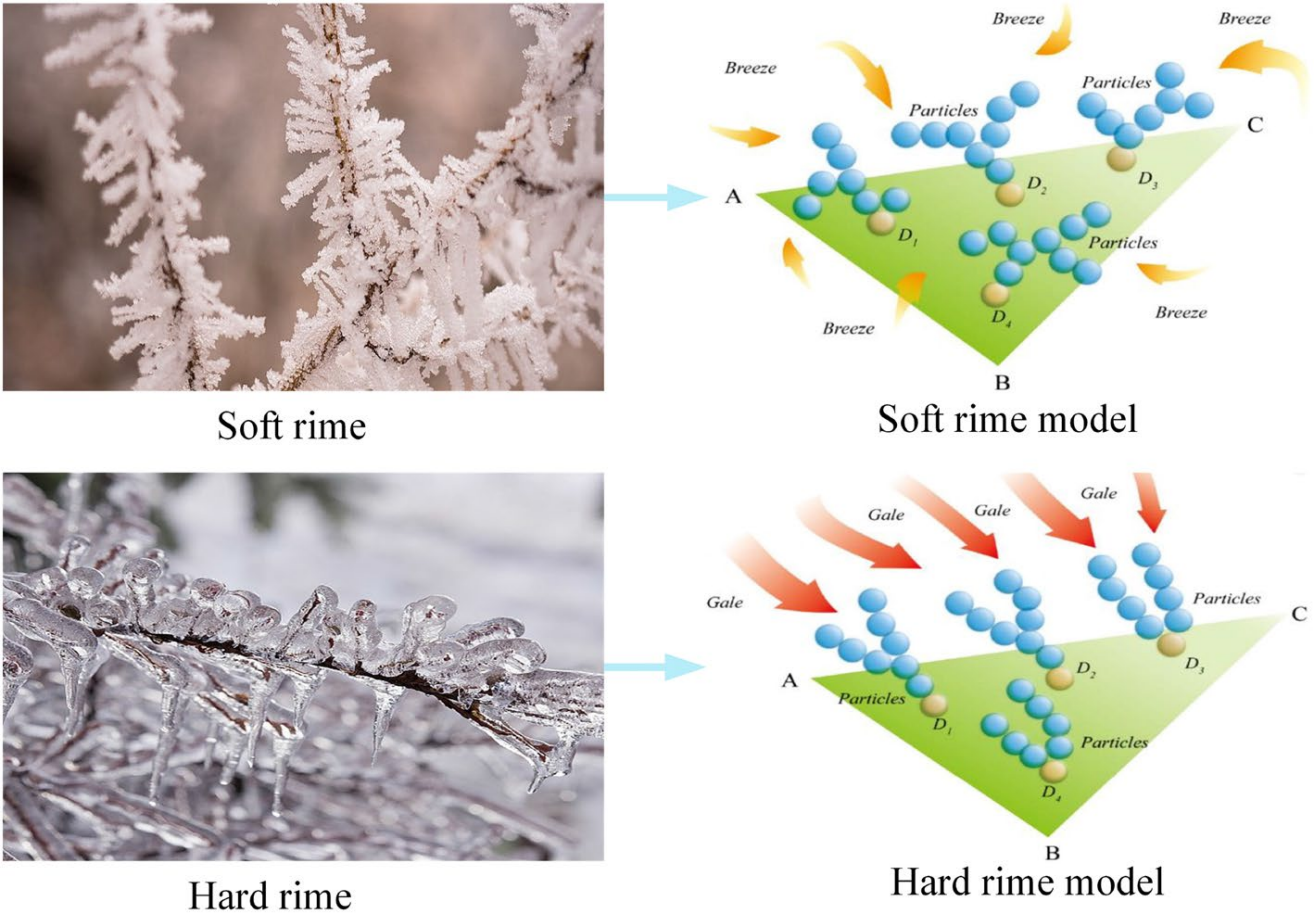
Two different forms of rime ice and their models^33^.

Fog ice forms through the freezing of water vapor in the air at low temperatures, influenced by factors such as temperature, wind speed, and humidity. In regions with unique climates and terrains, distinctive rime ice scenes may emerge. Rime ice typically comes in two forms: soft and hard, determined by wind speed. Soft rime develops under gentle breezes, growing slowly and irregularly, while strong winds lead to the formation of hard rime, characterized by rapid growth in a consistent direction. Figure 1 shows two different rime scenes and their corresponding mathematical models. Drawing inspiration from rime growth patterns, we propose the Soft Rime Search Strategy and Hard Rime Puncture Mechanism, alongside enhancements to the selection mechanism through positive greedy selection. By integrating these three mechanisms, the RIME algorithm is devised.

### Mathematical model of RIME

#### Population initialization

The initial population of rime is shown in Eq. 1.

1 $$\begin{array}{*{20}c} {R = \left[ {\begin{array}{*{20}c} {x_{{11}} } & {x_{{12}} } & \cdots & {x_{{1j}} } \\ {x_{{21}} } & {x_{{22}} } & \cdots & {x_{{2j}} } \\ \vdots & \vdots & \ddots & \vdots \\ {x_{{i1}} } & {x_{{i2}} } & \cdots & {x_{{ij}} } \\ \end{array} } \right]} \\ \end{array}$$

where $R$ is population matrix of rime, ${x}_{ij}$ denotes rime particle’s position.

#### Soft rime search strategy (exploration)

Soft rime grows randomly and covers a wide area in a breezy environment, but grows slowly in the same direction. Inspired by this, utilizing the random coverage of rime, a soft rime search strategy is proposed to make the algorithm quickly cover the search space at the beginning and avoid local optima. Its mathematical model is expressed as follows.

2 $$\begin{array}{c}{R}_{ij}^{new}={R}_{bestj}+{r}_{1}\times \text{cos}\left(\frac{t\times \pi }{10\times T}\right)\times \left(1-round\left(\frac{5\times t}{T}\right)\div 5\right)\times \left(h\times \left(U{b}_{ij}-L{b}_{ij}\right)+L{b}_{ij}\right),{r}_{2}<E\end{array}$$

where ${R}_{ij}^{new}$ is the rime particle position updated by the soft rime search strategy, ${R}_{bestj}$ represents the *jth* rime particle of the best individual in the population, ${r}_{1}$ denotes a random number between − 1 and 1, ${r}_{2}$ is a random number between 0 and 1, $round$ is a rounded integer, $h$ represents the adhesion between soft rime, $U{b}_{ij}$ and $L{b}_{ij}$ represent the upper and lower bounds of the search space respectively, $t$ is the current iteration number, $T$ denotes the maximum iteration number, and the attachment coefficient $E$ affects the condensation probability of the search individual and increases with the augment of iteration number, as shown in Eq. (3).

3 $$\begin{array}{c}E=\sqrt{\left(\frac{t}{T}\right)}\end{array}$$

#### Hard rime puncture mechanism (exploitation)

Hard rime grows more regularly in strong winds. Its characteristics are as follows: (1) The strong wind makes the hard rime grow rapidly in the unified direction. (2) Rime growing in the same direction are easy to cross, that is rime piercing. (3) Hard rime increases with growth, and the penetration probability is high when the growth condition is good. Therefore, inspired by the puncturing phenomenon, a hard rime puncturing mechanism is proposed to update the algorithm, realize particle exchange, and improve the convergence of the algorithm and the ability to jump out of local optimum. The particle replacement formula is given in Eq. (4).

4 $$\begin{array}{c}{R}_{ij}^{new}={R}_{best,j},{r}_{3}<{F}_{normr}\end{array}$$

where ${R}_{ij}^{new}$ is the rime particle position updated by the hard rime puncture mechanism, ${r}_{3}$ represent random number between 0 and 1, ${F}_{normr}$ denotes the current individual fitness value of normalized value.

#### Positive greedy selection mechanism

The metaheuristic algorithm often employs greedy mechanism to update both the global optimal solution and individual position. This involves comparing the performance of the updated individual with the global optimum. If the updated individual performs better, the global optimum is updated accordingly. While this approach is simple and fast, it solely focuses on recording the best results without necessarily enhancing individual search capabilities. To improve efficiency, a positive greedy mechanism is proposed to compare individual performance before and after updating, replacing the old individual with the updated one if it performs better. This practice encourages the emergence of superior individuals more frequently, thereby enhancing solution quality. Due to the change of individual position in the population, there may be worse agents than the population before updating, but it helps the population to develop in a better direction as a whole. The mathematical model is as follows:

5 $$\begin{array}{c}F\left({R}_{i}\right)=F\left({R}_{i}^{new}\right) {R}_{i}={R}_{i}^{new} , F\left({R}_{i}^{new}\right)<F\left({R}_{i}\right)\end{array}$$

6 $$\begin{array}{c}F({R}_{best})=F({R}_{i}^{new}) {R}_{best}={R}_{i}^{new} , F({R}_{i}^{new})<F({R}_{best})\end{array}$$

where $F\left({R}_{i}\right)$ represents the fitness value of the agent before updating, $F\left({R}_{i}^{new}\right)$ is the updated fitness value, $F({R}_{best})$ is the global optimal fitness value, ${R}_{i}$ represents the position before updating, ${R}_{i}^{new}$ is the updated position, and ${R}_{best}$ denotes the individual global optimal position.

**Figure 2:**
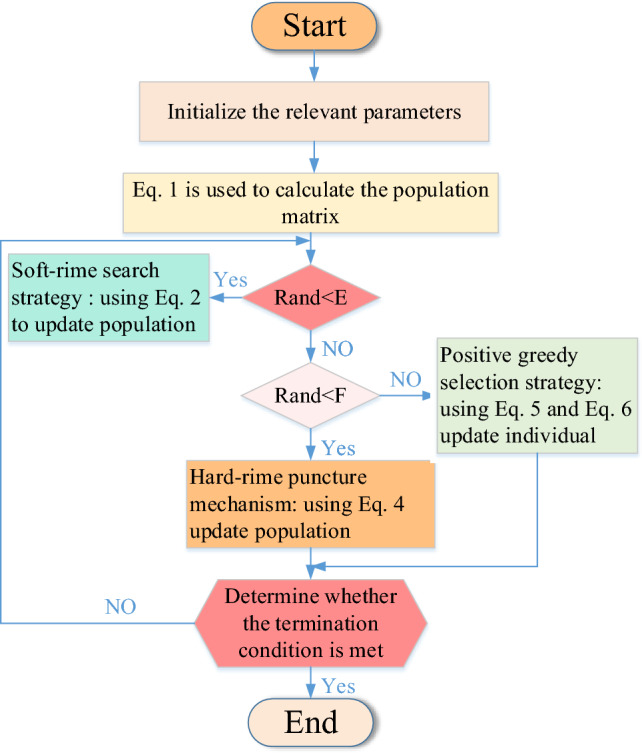
Flowchart of RIME.

In summary, the flowchart of RIME is shown in Fig. 2.

## The proposed MIRIME

This section describes our proposed MIRIME algorithm. Aiming at the problem that RIME has low accuracy and is easy to fall into local optimum in the experiment, we propose four strategies to improve it, and the specific content will be detailed in the subsequent sections.

### Population initialization based on tent chaotic map (TRIME)

The Tent map is a two-dimensional chaotic map known for its superior uniformity and ergodicity compared to pseudo-random number generation^31,38^. To address the issue of uneven distribution of rime particles initialized by the rime optimizer, the Tent map within the chaotic map is utilized to replace the pseudo-random method of rime particle initialization. This approach resolves the problem of uneven population initialization inherent in traditional methods, thereby enhancing the diversity of particle group searches and effectively mitigating the risk of particles falling into local optima. The specific formula of the Tent map is provided in Eq. (7):

7 $$\begin{array}{c}{x}_{n+1}=\left\{\begin{array}{c}\frac{{x}_{n}}{\alpha }{x}_{n}\in [0,\alpha )\\ \frac{1-{x}_{n}}{1-\alpha }{x}_{n}\in [\alpha ,1)\end{array}\right.\end{array}$$

where $\alpha$ is the value 1.1, ${x}_{n}$ is the matrix of the *n*^*th*^ mapping, and ${x}_{n+1}$ denotes the matrix of the *n*^*th*+1^ mapping.

### Adaptive update rule based on leader and dynamic centroid (ARIME)

In the soft rime search phase of the rime algorithm, frost ice particles are guided by the leader to explore in a more favorable direction. Simultaneously, considering the dynamic change of the centroid, individuals can cover the search space more comprehensively and avoid falling into local optima when they move toward the leader and the centroid. The presence of the leader enables the optimizer to progress towards the direction currently deemed optimal, while the dynamic centroid serves as a reference point reflecting the central tendency of the group. This facilitates maintaining synergy with the group during the exploration process. This combined approach enables the algorithm to extensively explore the solution space initially and uncover more potential optimal solutions. In the iterative process, it gradually focuses on the high-potential area, effectively balancing the relationship between exploration and exploitation^39^. The mathematical model is as follows:

8 $$\begin{array}{c}{R}_{ij}^{new}={R}_{bestj}+{X}_{m}-{R}_{ij}\end{array}$$

Where ${R}_{ij}^{new}$ is the rime particle position after the soft rime search strategy update, ${R}_{ij}$ represents the rime particle position before update, ${R}_{bestj}$ denotes the *jth* rime particle of the best individual in the population, and ${X}_{m}$ shows the average of the positions of the former $A$ agents, as reported in Eq. (9).

9 $$\begin{array}{c}{X}_{m}=mean\left({R}_{A}\right)\end{array}$$

where ${R}_{A}$ on behalf of former *A* agent location. *A* is 2 to *N* random integers, *N* denotes rime populations.

### Control strategy based on lens opposition-based learning (LRIME)

**Figure 3:**
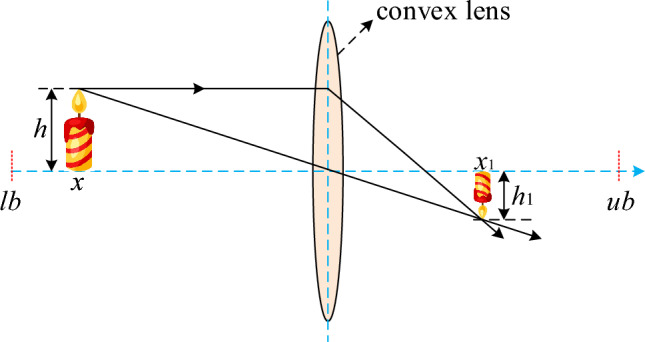
Opposition-based learning model for lens imaging.

In order to solve the problem of low convergence accuracy caused by the decrease of population diversity in the later iteration of RIME, the lens imaging opposition-based learning mechanism is introduced to solve this shortcoming, so as to improve the optimization ability of the algorithm^40,41^. The lens imaging opposition-based learning model is shown in Fig. 3.

Where the $lb$, $ub$ respectively for the lower bound and upper bound, $h$ and $h$ **1** respectively represent the original height and through the height of the lens. The mathematical model is as follows:

10 $$\begin{array}{c}\frac{(lb+ub)/2-x}{x1-(lb+ub)/2}=\frac{h}{{h}_{1}}\end{array}$$

where *x* and $x1$ represent the projection points of the object, assuming $k=\frac{h}{{h}_{1}}$, Eq. (16) can be simplified as follows:

11 $$\begin{array}{c}x1=\frac{lb+ub}{2}+\frac{lb+ub}{2\times k}-\frac{x}{k}\end{array}$$

When the $k=1$, becomes reverse learning strategy. This article take $k$ mathematical expression is as follows:

12 $$\begin{array}{c}k={\left(1+{\left(\frac{t}{T}\right)}^{0.5}\right)}^{10}\end{array}$$

### Centroid boundary control strategy (CRIME)

The typical idea of the traditional boundary control strategy is to compare the updated position of the agent with the upper and lower boundaries. If the updated position exceeds the boundary, the current position is replaced with the boundary value^42^. While this method prevents individuals from existing beyond the boundary, it does not contribute to the search performance of the optimizer. To address this limitation, we propose a centroid boundary control mechanism to participate in population updating, thereby enhancing global exploration efficiency. Specifically, by constraining the search boundary of individuals and utilizing the group centroid as the guiding point, the search focus and efficiency of the optimization algorithm are significantly improved. This strategy not only prevents individuals from exploring invalid regions and minimizes resource wastage but also ensures the concentration of the group in high-potential areas, facilitating the discovery of high-quality solutions. It enhances the adaptability and robustness of the optimizer to the optimization problem, thereby playing a pivotal role in improving solution quality and accelerating convergence speed. The mathematical model is given in Eqs. (13) and (14).

13 $$\begin{array}{c}{R}_{ij}^{new}=\left(mean\left(R\right)+U{b}_{ij}\right)\div 2\end{array}$$

14 $$\begin{array}{c}{R}_{ij}^{new}=\left(mean\left(R\right)+L{b}_{ij}\right)\div 2\end{array}$$

where $mean\left(R\right)$ is search agent location of the mean.

**Figure 4:**
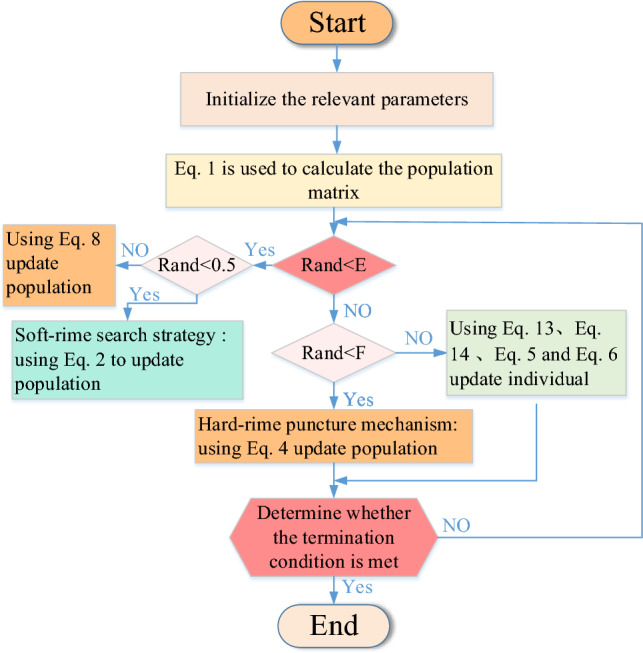
Flowchart of MIRIME.

In summary, the flowchart of MIRIME is shown in Fig. 4. The pseudocode is reported in Algorithm 1.

**Algorithm 1:**
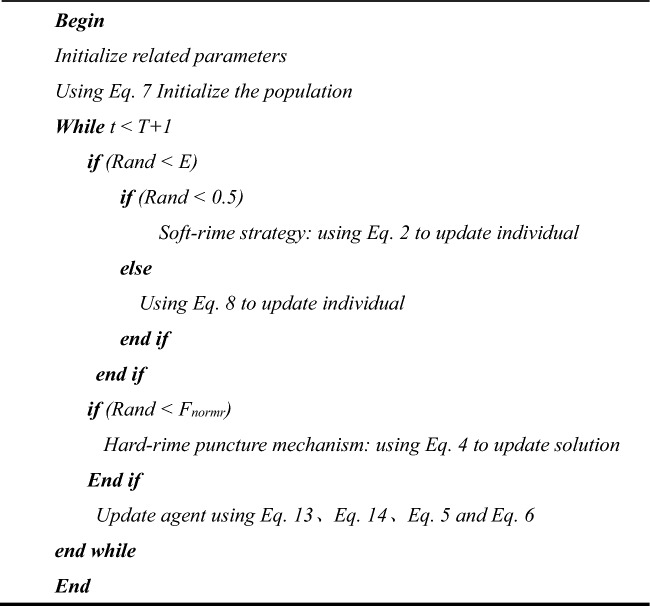
Pseudo-Code of MIRIME Algorithm.

### Time complexity analysis

The computational complexity of algorithm is a key indicator to measure the execution time of the algorithm^43,44^. In this paper, we apply the Big $O$ complexity notation to analyze the time complexity of the RIME and MIRIME algorithms. The complexity of the soft rime search strategy is $O\left({N}^{2}\right)$, $N$ represents the number of rime populations, the complexity of the hard rime puncture mechanism is $O\left({N}^{2}\right)$, the complexity of the positive greedy selection mechanism is $O\left(N\right)$, and the complexity of calculating the fitness value is $O\left(N\times log(N)\right)$. Therefore, the total time complexity is $O\left(\left(N+log\left(N\right)\right)\times N\right)$, because MIRIME did not introduce a new loop, the time complexity is $O\left(\text{MIRIME}\right)=O\left(\text{RIME}\right)=O\left(\left(N+log\left(N\right)\right)\times N\right)$.

## Analysis of experimental results

In this section, we comprehensively evaluate the performance of the MIRIME algorithm on CEC 2017. Experiments were designed to analyze its convergence, exploration and exploitation ability, population diversity, and other aspects, and compare it with 11 advanced algorithms. The Wilcoxon and Friedman nonparametric tests are used to analyze the performance differences among the algorithms. The experimental equipment used is a laptop with an Intel Core i5-12500H, 2.5 GHz CPU, and 16 GB RAM, running MATLAB 2023a. Through this rigorous series of experiments, we provide strong support for the performance of the MIRIME optimizer.

### Benchmark functions

The benchmark test function serves as a crucial tool for evaluating the performance of algorithms, offering a standardized platform to assess and compare various optimization optimizers^45^. In this study, we utilize the CEC2017 test suite to evaluate the performance of the proposed MIRIME algorithm across dimensions of 10, 30, and 50, respectively. With increasing dimensionality, the number of local optimal solutions also increases, enabling the suite to effectively evaluate the algorithm's global optimization capability^46^. For further details regarding CEC2017, please refer to Table 1. Additionally, to further validate the optimization capabilities of the algorithm, we utilize the CEC2022 test set to evaluate the performance of the MIRIME algorithm, with dimensions set at 10 and 20 respectively. The specific details of CEC2022 are detailed in Table 2.

**Table 1:** The CEC2017 test suite

Type	ID	CEC2017 Function name	Rang	Dimension	*f*_min_
Unimodal	F1	Shifted and rotated Bent Cigar function	[− 100,100]	10/30/50	100
	F2	Shifted and rotated sum of different power function	[− 100,100]	10/30/50	200
	F3	Shifted and rotated Zakharov Function	[− 100,100]	10/30/50	300
Multimodal	F4	Shifted and rotated Rosenbrock’s Function	[− 100,100]	10/30/50	400
	F5	Shifted and Rotated Rastrigin’s Function	[− 100,100]	10/30/50	500
	F6	Shifted and Rotated Expanded Scaffer’s F6 function	[− 100,100]	10/30/50	600
	F7	Shifted and rotated Lunacek Bi_Rastrigin function	[− 100,100]	10/30/50	700
	F8	Shifted and rotated non-continuous Rastrigin’s function	[− 100,100]	10/30/50	800
	F9	Shifted and rotated levy function	[− 100,100]	10/30/50	900
	F10	Shifted and rotated Schwefel’s function	[− 100,100]	10/30/50	1000
Hybrid	F11	Hybrid function 1 (N = 3)	[− 100,100]	10/30/50	1100
	F12	Hybrid function 2 (N = 3)	[− 100,100]	10/30/50	1200
	F13	Hybrid function 3 (N = 3)	[− 100,100]	10/30/50	1300
	F14	Hybrid function 4 (N = 4)	[− 100,100]	10/30/50	1400
	F15	Hybrid function 5 (N = 4)	[− 100,100]	10/30/50	1500
	F16	Hybrid function 6 (N = 4)	[− 100,100]	10/30/50	1600
	F17	Hybrid function 6 (N = 5)	[− 100,100]	10/30/50	1700
	F18	Hybrid function 6 (N = 5)	[− 100,100]	10/30/50	1800
	F19	Hybrid function 6 (N = 5)	[− 100,100]	10/30/50	1900
	F20	Hybrid function 6 (N = 6)	[− 100,100]	10/30/50	2000
Composition	F21	Composition function 1 (N = 3)	[− 100,100]	10/30/50	2100
	F22	Composition function 2 (N = 3)	[− 100,100]	10/30/50	2200
	F23	Composition Function 3 (N = 4)	[− 100,100]	10/30/50	2300
	F24	Composition function 4 (N = 4)	[− 100,100]	10/30/50	2400
	F25	Composition function 5 (N = 5)	[− 100,100]	10/30/50	2500
	F26	Composition function 6 (N = 5)	[− 100,100]	10/30/50	2600
	F27	Composition function 7 (N = 6)	[− 100,100]	10/30/50	2700
	F28	Composition function 8 (N = 6)	[− 100,100]	10/30/50	2800
	F29	Composition function 9 (N = 3)	[− 100,100]	10/30/50	2900
	F30	Composition function 10 (N = 3)	[− 100,100]	10/30/50	3000

**Table 2:** The CEC2022 test suite

Type	ID	CEC2022 function name	Rang	Dimension	*f*_min_
Unimodal	F1	Shifted and full rotated Zakharov function	[− 100,100]	10/20	100
Multimodal	F2	Shifted and full rotated Rosenbrock’s function	[− 100,100]	10/20	200
	F3	Shifted and full rotated Rastrigin’s function	[− 100,100]	10/20	300
	F4	Shifted and full rotated non-continuous Rastrigin’s function	[− 100,100]	10/20	400
	F5	Shifted and full rotated Levy function	[− 100,100]	10/20	500
Hybrid	F6	Hybrid function 1 (N = 3)	[− 100,100]	10/20	600
	F7	Hybrid function 2 (N = 6)	[− 100,100]	10/20	700
	F8	Hybrid function 3 (N = 5)	[− 100,100]	10/20	800
Composition	F9	Composition function 1 (N = 5)	[− 100,100]	10/20	900
	F10	Composition function 2 (N = 4)	[− 100,100]	10/20	1000
	F11	Composition function 3 (N = 5)	[− 100,100]	10/20	1100
	F12	Composition function 4 (N = 6)	[− 100,100]	10/20	1200

### Parameter setting of competitor algorithm

MIRIME is compared with 11 other swarm intelligence optimization algorithms, including PO^47^, HLOA^48^, LEA^49^, HEOA^50^, NRBO^23^, MELGWO^51^, HPHHO^52^, PPSO^53^, EWOA^54^, SRIME^55^, and RIME^33^. Table 3 presents the parameter settings of these optimizers. The maximum number of iterations and population size are set to 500 and 30, respectively, and each algorithm is run independently 30 times. The maximum number of evaluations is 30,000. Subsequently, the standard deviation (Std) and average value (Ave) are calculated, reflecting the convergence speed and robustness of the algorithm, respectively^56^. The best results are highlighted in bold.

**Table 3:** Parameter Settings for the selected competitive algorithm

Algorithms	Name of the parameter	Value of the parameter
PO	St	1, 2, 3, 4
HLOA	Vo, g	1, 0.009807
LEA	h-max, h-min	0.7, 0
HEOA	A, LN, FN, EN	0.6, 0.4, 0.1, 0.4
NRBO	DF	0.6
MELGWO	Fmin, Fmax	0.1, 2
HPHHO	CR, w, F	0.9, 4, 0.5
PPSO	θ	[− 1, 1]
EWOA	P-rate	20
SRIME	w, r	5, (0, 1)
RIME	w, r	5, (0, 1)
MIRIME	w, r	5, (0, 1)

### Qualitative analysis

In this section, we provide a qualitative analysis of the MIRIME algorithm, focusing on three aspects of the experimental design: population diversity, exploration and exploitation, and convergence behavior.

#### Exploration and exploitation analysis

The search process of metaheuristics mainly consists of two phases: exploration and exploitation. The exploration stage seeks multiple possible solutions to the problem, while the exploitation phase relies on local information to optimize the schemes. Balancing these two stages is crucial, as long-term exploration may lead to slow convergence of the algorithm, while overexploitation may fall into local optima^57,58^. We use Eqs. (15) and (16) to calculate the percentages of exploration and exploitation, respectively. $Div(t)$ is a measure of diversity and is calculated by Eq. (17). Where ${x}_{id}$ is the position of the *i*^*th*^ agent and ${Div}_{max}$ denotes the maximum diversity during the iteration^59^.

15 $$\begin{array}{c}Exploration\left(\%\right)=\frac{Div\left(t\right)}{{Div}_{max}}\times 100\end{array}$$

16 $$\begin{array}{c}Exploitation\left(\%\right)=\frac{\left|Div\left(t\right)-{Div}_{max}\right|}{{Div}_{max}}\times 100\end{array}$$

17 $$\begin{array}{c}Div\left(t\right)=\frac{1}{D}\sum_{d=1}^{D}\frac{1}{N}\sum_{i=1}^{N}\left|median\left({x}_{d}\left(t\right)\right)-{x}_{id}\left(t\right)\right|\end{array}$$

**Figure 5:**
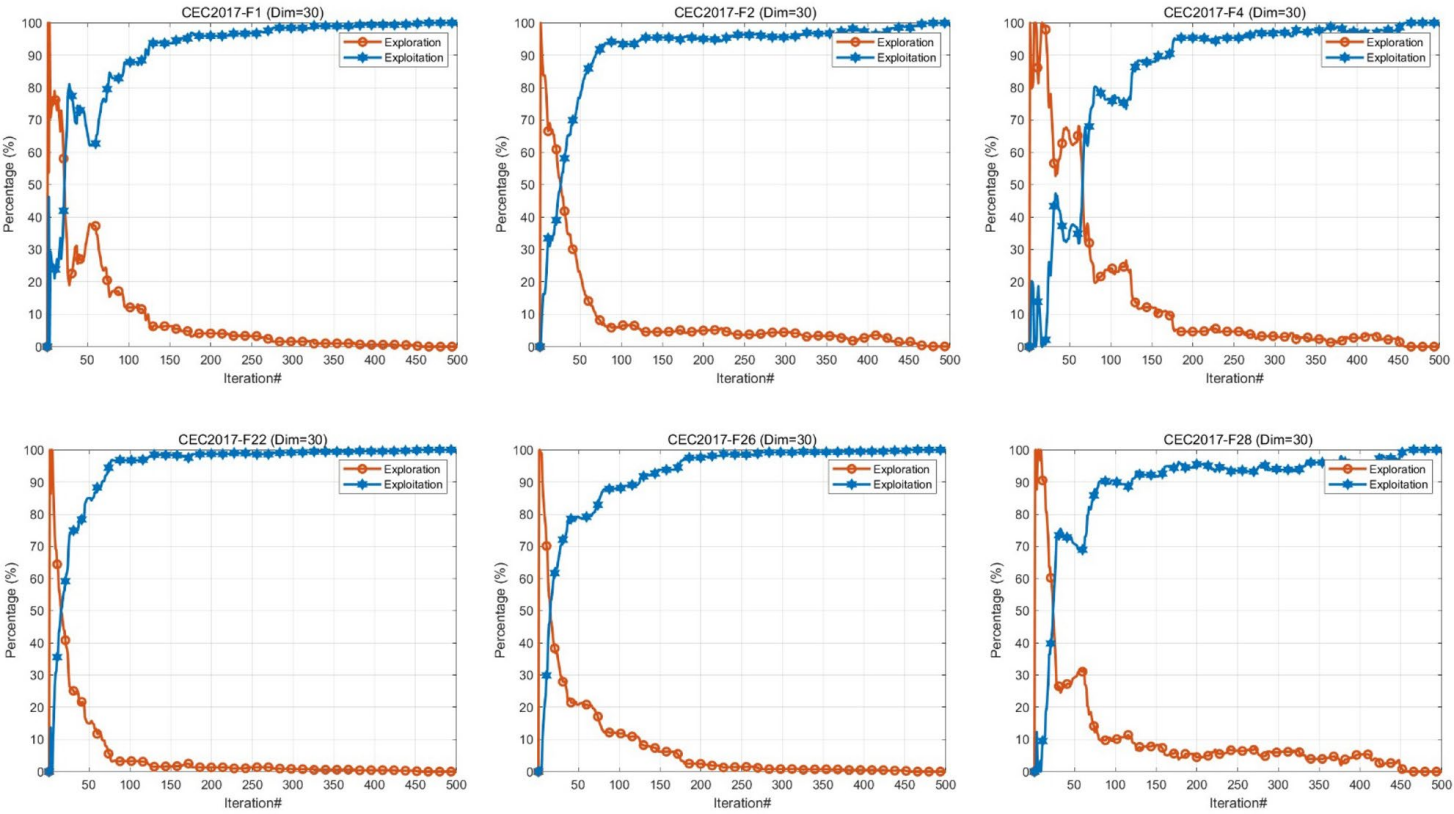
Exploration and exploitation of MIRIME.

Figure 5 depicts the experimental results of MIRIME on CEC2017 with 30 dimensions. The horizontal axis represents the number of iterations, while the vertical axis indicates the percentage of exploration versus exploitation. MIRIME achieves a balanced ratio between exploration and exploitation throughout the iteration process. It explores extensively during the early stages, showcasing excellent exploration capabilities. As the iteration progresses, it maintains a higher proportion of exploitation to enhance convergence speed and accuracy. Throughout the entire iterative process, MIRIME maintains a dynamic equilibrium between exploration and exploitation, effectively mitigating the risk of local optima and premature convergence.

#### Population diversity analysis

In swarm intelligence algorithms, population diversity is extremely important. High population diversity means that the algorithm can explore more widely in the search space, thereby reducing the risk of falling into the local optimal solutions prematurely^60^. To evaluate the differences between RIME and MIRIME in terms of population diversity, we conducted population diversity experiments on the CEC2017 test set with dimension 30. The population diversity is calculated by the moment of inertia ${I}_{C}$, which reflects the degree of dispersion of the population members relative to the centroid $c$, as shown in Eq. (18), where ${c}_{d}$ is the dispersion of the population members. As described in Eq. (19), where ${x}_{id}\left(t\right)$ represents the value of the search agent at the *t*^*th*^ iteration^61^.

18 $$\begin{array}{c}{I}_{C}\left(t\right)=\sqrt{\sum_{i=1}^{N}\sum_{d=1}^{D}{\left({x}_{id}\left(t\right)-{c}_{d}\left(t\right)\right)}^{2}}\end{array}$$

19 $$\begin{array}{c}{c}_{d}\left(t\right)=\frac{1}{D}\sum_{i=1}^{N}{x}_{id}\left(t\right)\end{array}$$

**Figure 6:**
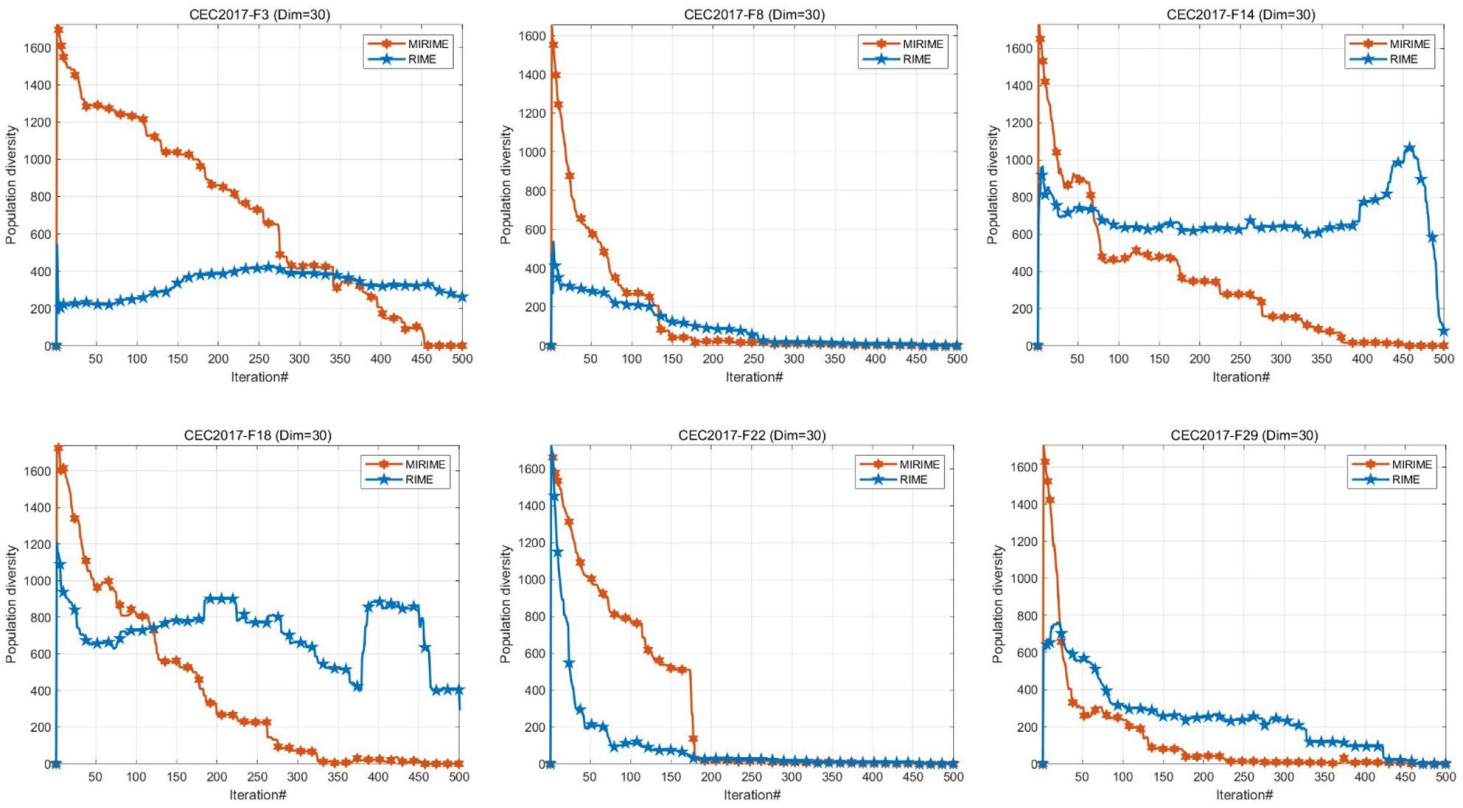
Population diversity between RIME and MI RIME.

The experimental results are depicted in Fig. 6. It is evident that MIRIME exhibits stronger exploitation performance compared to RIME throughout the entire iteration process. This indicates that the enhanced optimizer can more effectively explore the search space and mitigate issues such as premature convergence and local stagnation. Consequently, MIRIME demonstrates higher potential in discovering local optimal solutions.

#### Convergence behavior analysis of the MIRIME

**Figure 7:**
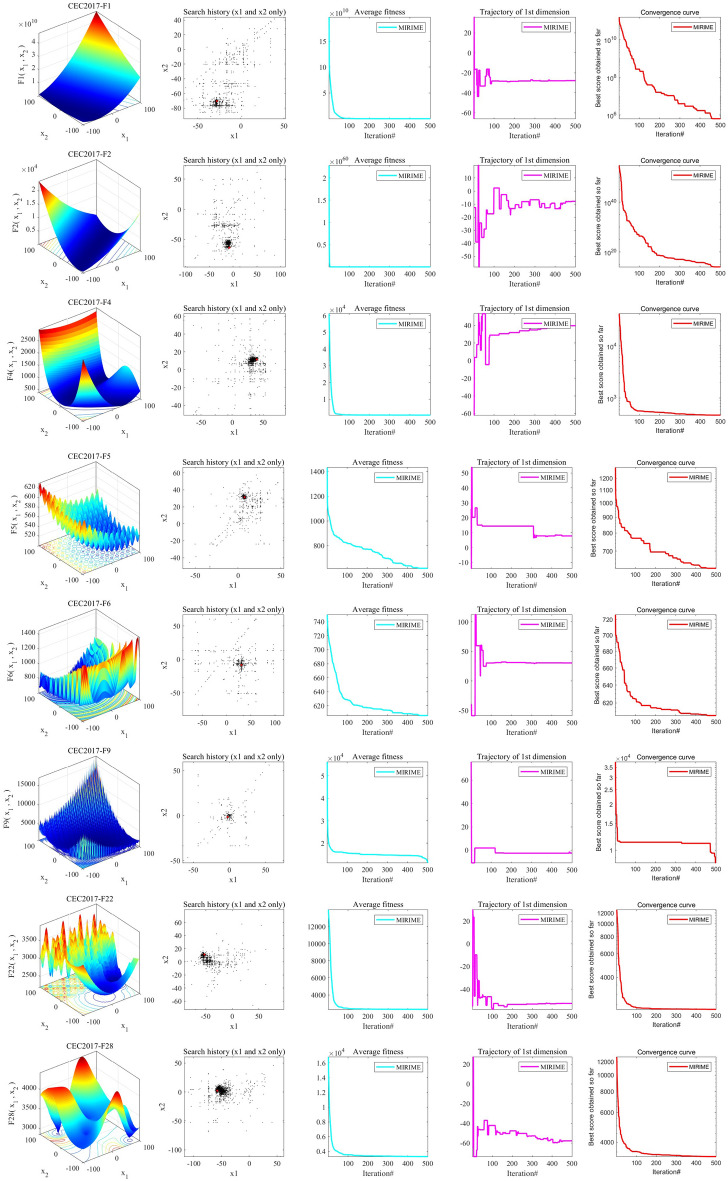
Convergence behavior of MIRIME.

To assess the convergence of the MIRIME algorithm, we conduct experiments and analyze its convergence behavior. Figure 7 presents the experimental results. The first column clearly depicts the search space of the test function. The red dot in the second column signifies the global optimal solution, while the black dots denote the locations of the search agents. It's noteworthy that most individuals are distributed near the optimal solution, indicating that MIRIME effectively explores the search space and avoids local stagnation. The third column illustrates the average fitness value change of the search agents. A high initial stage reflects extensive exploration, followed by a rapid decline, indicating that most search agents have the potential to find the best value. The fourth column displays the search trajectory of the individual, from initial fluctuation to later stability, showing the transition from extensive exploration to local exploitation^62^. The last column presents the convergence curve for MIRIME. The curve for unimodal functions steadily decreases, signifying that the algorithm converges to the optimal value with a sufficient number of iterations. For multi-modal functions, the curve decreases gradually, demonstrating that the optimizer can consistently escape local optima to reach the global optimum.

### Quantitative analysis

In this section, we utilize the CEC 2017 and CEC2022 test suite to evaluate the effectiveness of MIRIME.

#### Comparison with other competitive algorithms in CEC 2017

**Figure 8:**
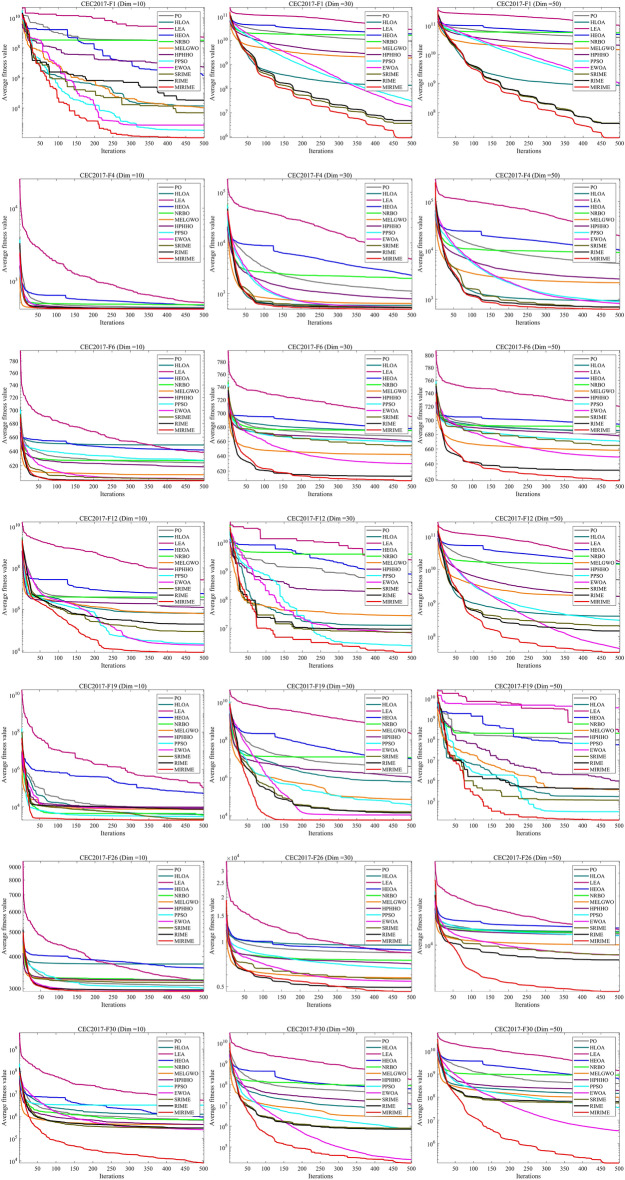
Comparison of convergence curves of 12 algorithms on CEC2017.

**Figure 9:**
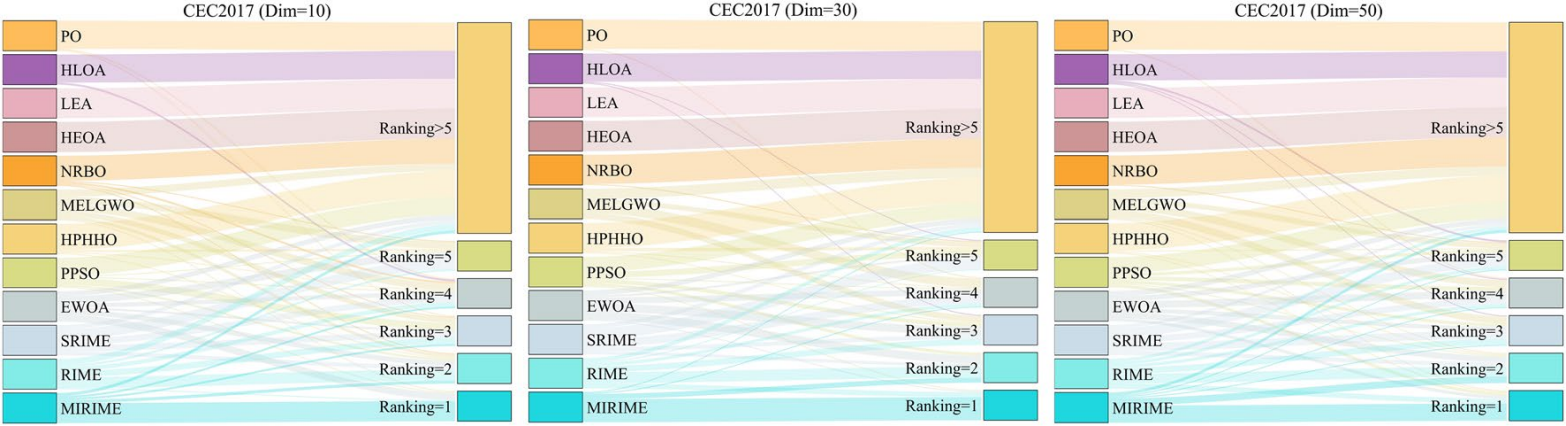
The ranking Sankey of different competitors on CEC2017.

**Figure 10:**
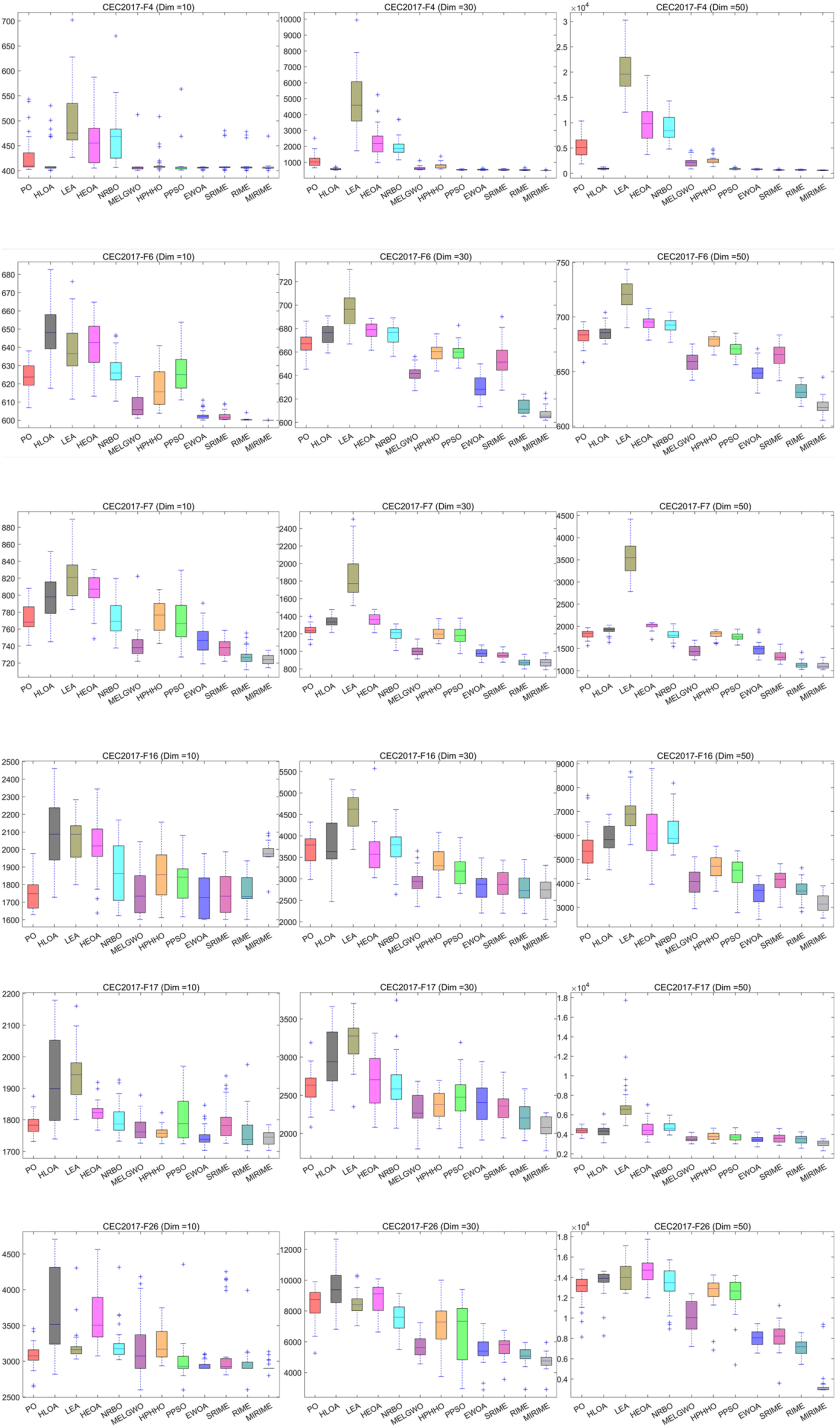
Boxplot analysis of competitor algorithms on the CEC2017 test suite.

To demonstrate the competitiveness of our proposed MIRIME optimizer, we conducted performance tests on three different dimensions: 10, 30, and 50 using the CEC 2017 suite. The experimental results are summarized in Tables 4, 5, and 6. The results indicate that with the increase in problem dimension, the performance of other algorithms is greatly affected and they are prone to falling into local optima, whereas MIRIME exhibits better stability and robustness. In the last line of the table, three symbols (W|T| L) are used to mark the performance of MIRIME on each test function compared with other algorithms, representing the number of wins (W), ties (T), and losses (L) compared to other algorithms. It is worth noting that the number of wins (W) refers to the number ranked first by the Friedman test, and the number of losses (L) refers to the number ranked last. The experimental data show that MIRIME exhibits a high number of wins in all tested dimensions, with no underperformance. Figure 8 illustrates the convergence curves of different algorithms in each dimension. As the dimension increases, optimization becomes more challenging, and other algorithms are prone to falling into local optima. In contrast, MIRIME's convergence curves mostly show a continuous downward trend, consistently maintaining a lower trajectory than that of other algorithms, demonstrating its strong potential in finding the optimal solution and overall outperforming the competing algorithms. Figure 9 displays the Friedman ranking of MIRIME and its comparison algorithms in 10, 30, and 50 dimensions of the CEC2017 test set with a Sankey diagram. In the Sankey diagram, it is obvious that our proposed MIRIME predominantly targets the highest-ranked color region, whereas the other algorithms predominantly occupy the lower-ranked color regions. Consequently, this demonstrates that MIRIME secures the highest number of first-place rankings across all three dimensions. In Fig. 10, the performance of the 12 algorithms on three different dimensions of the CEC2017 test set is presented in detail with box plots, In the box plots, it is apparent that the MIRIME algorithm maintains a lower box position compared to its competitors, suggesting superior optimization values. Additionally, its bin width is the narrowest, indicating that MIRIME achieves data consistency with minimal fluctuations and enhanced stability. Consequently, these factors demonstrate that MIRIME delivers the best overall performance. This fully demonstrates the excellent performance of the MIRIME algorithm in global exploration and local exploitation, verifying its effectiveness and accuracy.

**Table 4:** Experimental results of 12 algorithms on CEC 2017 (Dim = 10)

ID		PO	HLOA	LEA	HEOA	NRBO	MELGWO	HPHHO	PPSO	EWOA	SRIME	RIME	MIRIME
F1	Ave	3.1064E+07	1.4854E+05	5.9704E+08	3.5414E+08	5.9239E+08	3.9296E+03	3.7816E+06	2.7553E+03	**2.1335E+03**	1.9381E+04	2.2216E+04	2.3111E+03
	Std	7.6378E+07	3.3484E+05	3.1429E+08	3.7250E+08	6.0665E+08	3.8217E+03	5.0230E+06	2.5180E+03	**2.0011E+03**	1.6536E+04	1.4874E+04	2.7712E+03
F2	Ave	6.8741E+06	1.5004E+06	1.7737E+10	5.4638E+07	5.4858E+07	2.0031E+03	1.2480E+05	5.2204E+03	4.9214E+03	3.0690E+02	5.2213E+02	**2.0070E+02**
	Std	1.3273E+07	4.4683E+06	4.8261E+10	8.7640E+07	1.4367E+08	4.7474E+03	1.7070E+05	8.0466E+03	8.5169E+03	3.0214E+02	5.4329E+02	**3.8341E+00**
F3	Ave	1.0653E+03	3.4348E+02	2.6954E+04	5.1585E+03	1.8492E+03	3.0819E+02	9.8627E+02	4.4374E+02	9.7997E+02	3.0137E+02	3.0157E+02	**3.0007E+02**
	Std	1.3607E+03	4.8732E+01	1.0745E+04	2.1895E+03	1.2066E+03	3.3036E+01	6.4328E+02	7.7632E+02	5.8724E+02	1.1317E+00	1.1346E+00	**9.1973E-02**
F4	Ave	4.3007E+02	4.2227E+02	4.9959E+02	4.6070E+02	4.6794E+02	4.0877E+02	4.1437E+02	4.1243E+02	**4.0553E+02**	4.1288E+02	4.1463E+02	4.0842E+02
	Std	3.9283E+01	3.4593E+01	6.1169E+01	4.7221E+01	5.3039E+01	1.9682E+01	2.1146E+01	3.0910E+01	**2.0224E+00**	2.0792E+01	2.2909E+01	1.1715E+01
F5	Ave	5.4646E+02	5.7365E+02	5.7091E+02	5.6659E+02	5.4559E+02	5.2226E+02	5.3650E+02	5.4243E+02	5.2726E+02	5.3220E+02	5.1782E+02	**5.1619E+02**
	Std	1.8304E+01	2.8618E+01	1.9327E+01	1.9290E+01	1.3727E+01	8.5487E+00	1.2160E+01	1.8143E+01	1.0574E+01	1.3049E+01	7.6898E+00	**5.9618E+00**
F6	Ave	6.2390E+02	6.4883E+02	6.3843E+02	6.4177E+02	6.2681E+02	6.0791E+02	6.1847E+02	6.2734E+02	6.0276E+02	6.0234E+02	6.0036E+02	**6.0002E+02**
	Std	7.1707E+00	1.4776E+01	1.5200E+01	1.2801E+01	8.7091E+00	6.5747E+00	1.0388E+01	1.1917E+01	2.6792E+00	2.3852E+00	7.5099E−01	**1.3467E−02**
F7	Ave	7.7181E+02	7.9655E+02	8.2142E+02	8.0419E+02	7.7253E+02	7.4155E+02	7.7452E+02	7.6932E+02	7.4705E+02	7.3800E+02	7.2809E+02	**7.2368E+02**
	Std	1.7496E+01	2.7858E+01	2.5722E+01	1.9555E+01	2.0845E+01	1.8247E+01	1.7300E+01	2.5491E+01	1.7500E+01	9.9945E+00	9.7727E+00	**5.7042E+00**
F8	Ave	8.2998E+02	8.4715E+02	8.7030E+02	8.3893E+02	8.3670E+02	8.1841E+02	8.2843E+02	8.3478E+02	8.2375E+02	8.2815E+02	8.1737E+02	**8.1708E+02**
	Std	6.6506E+00	2.2039E+01	1.7992E+01	8.7783E+00	1.0658E+01	6.0081E+00	6.3327E+00	1.4188E+01	7.7499E+00	1.4480E+01	8.0810E+00	**5.2469E+00**
F9	Ave	1.1158E+03	1.5584E+03	2.2880E+03	1.4622E+03	1.0890E+03	9.4440E+02	1.1760E+03	1.1500E+03	9.4973E+02	9.0094E+02	9.0050E+02	**9.0004E+02**
	Std	1.7936E+02	2.8200E+02	7.2752E+02	2.8179E+02	1.5473E+02	4.0406E+01	2.1479E+02	1.5074E+02	4.9456E+01	1.8636E+00	1.6769E+00	**1.2746E−01**
F10	Ave	2.0453E+03	2.3760E+03	2.7724E+03	2.1822E+03	2.2399E+03	1.7576E+03	1.9056E+03	2.1095E+03	1.8795E+03	1.7635E+03	1.5824E+03	**1.4807E+03**
	Std	3.1659E+02	3.7251E+02	3.9873E+02	2.5490E+02	3.6908E+02	2.8257E+02	3.2277E+02	3.0669E+02	2.9990E+02	2.9029E+02	2.2269E+02	**1.8582E+02**
F11	Ave	1.1980E+03	1.1905E+03	1.6608E+03	1.2560E+03	1.2311E+03	1.1549E+03	1.1671E+03	1.1518E+03	1.1279E+03	1.1284E+03	1.1184E+03	**1.1078E+03**
	Std	7.8235E+01	6.2169E+01	4.3786E+02	1.0528E+02	9.0828E+01	3.4947E+01	5.7038E+01	3.1277E+01	2.6435E+01	2.5071E+01	3.0725E+01	**3.5531E+00**
F12	Ave	2.8822E+06	6.5236E+05	2.6383E+07	5.7635E+06	3.9191E+06	6.3166E+05	1.2413E+06	2.1914E+04	1.9067E+04	8.5878E+04	1.9536E+05	**8.5224E+03**
	Std	4.1707E+06	1.0329E+06	2.6545E+07	3.5038E+06	3.9243E+06	7.5530E+05	1.3437E+06	1.5145E+04	1.5579E+04	1.0249E+05	3.9652E+05	**4.4411E+03**
F13	Ave	1.4395E+04	7.8080E+03	3.9303E+05	1.3799E+04	6.1593E+03	7.9174E+03	1.2048E+04	**1.7981E+03**	1.1253E+04	8.7093E+03	1.3088E+04	9.4022E+03
	Std	1.0017E+04	7.4300E+03	4.2186E+05	7.8385E+03	5.9589E+03	6.4278E+03	9.7461E+03	**5.0512E+02**	8.5651E+03	9.3741E+03	1.0201E+04	5.4029E+03
F14	Ave	2.5132E+03	2.4882E+03	1.1501E+04	2.0266E+03	1.5134E+03	1.6739E+03	1.5308E+03	**1.4589E+03**	1.5935E+03	1.6599E+03	4.4107E+03	1.6388E+03
	Std	1.4393E+03	4.9630E+03	1.1661E+04	1.7476E+03	4.4319E+01	4.1927E+02	6.4580E+01	**3.0140E+01**	2.4236E+02	4.6599E+02	3.7592E+03	8.1374E+02
F15	Ave	3.2547E+03	5.1170E+03	6.3539E+04	1.0040E+04	2.1600E+03	3.6194E+03	2.2040E+03	**1.6234E+03**	2.6707E+03	1.8272E+03	6.9293E+03	1.6509E+03
	Std	1.6921E+03	1.2768E+04	7.2409E+04	2.7361E+03	7.2190E+02	2.8757E+03	7.2258E+02	**8.2470E+01**	1.3703E+03	9.7514E+02	6.4304E+03	4.2030E+02
F16	Ave	1.7501E+03	2.0772E+03	2.0690E+03	2.0249E+03	1.8688E+03	1.7619E+03	1.8547E+03	1.8228E+03	**1.7269E+03**	1.7521E+03	1.7379E+03	1.9797E+03
	Std	8.7100E+01	2.0199E+02	1.3214E+02	1.6161E+02	1.6789E+02	1.3000E+02	1.4107E+02	1.2503E+02	1.1410E+02	1.2138E+02	9.6371E+01	**5.4861E+01**
F17	Ave	1.7864E+03	1.9319E+03	1.9357E+03	1.8225E+03	1.8024E+03	1.7710E+03	1.7606E+03	1.8044E+03	1.7470E+03	1.7968E+03	1.7578E+03	**1.7438E+03**
	Std	3.1483E+01	1.3355E+02	7.9606E+01	3.4270E+01	4.9255E+01	3.5438E+01	**2.1604E+01**	6.8619E+01	3.0672E+01	5.9195E+01	6.0278E+01	2.2406E+01
F18	Ave	2.1967E+04	1.9152E+04	2.3747E+06	2.8914E+04	1.0273E+04	1.7920E+04	1.3940E+04	5.3867E+03	1.4408E+04	1.2836E+04	1.0707E+04	**4.1394E+03**
	Std	1.3921E+04	1.5466E+04	4.3306E+06	1.2980E+04	9.6618E+03	1.2725E+04	1.1597E+04	**4.9004E+03**	9.1626E+03	1.1040E+04	7.9697E+03	5.8455E+03
F19	Ave	8.3110E+03	3.8815E+03	1.0792E+05	5.0661E+04	3.4763E+03	7.0511E+03	9.3664E+03	2.9861E+03	7.9531E+03	2.1884E+03	7.4914E+03	**1.9258E+03**
	Std	9.6805E+03	4.7796E+03	1.4604E+05	1.1583E+05	3.6055E+03	7.0662E+03	9.0513E+03	5.6569E+03	7.1885E+03	9.7154E+02	5.7513E+03	**7.5559E+01**
F20	Ave	2.1335E+03	2.2759E+03	2.1951E+03	2.2225E+03	2.1640E+03	2.1041E+03	2.1142E+03	2.1167E+03	2.0496E+03	2.1125E+03	**2.0490E+03**	2.0918E+03
	Std	5.2505E+01	1.0684E+02	8.1400E+01	5.4584E+01	7.7187E+01	5.7896E+01	5.8970E+01	6.8716E+01	**3.4488E+01**	7.3549E+01	5.7245E+01	5.5951E+01
F21	Ave	2.2569E+03	2.3528E+03	2.3313E+03	2.3340E+03	2.3225E+03	2.2979E+03	2.2286E+03	2.2613E+03	**2.2042E+03**	2.3126E+03	2.3081E+03	2.3049E+03
	Std	5.5067E+01	4.9204E+01	5.7409E+01	4.2015E+01	5.3609E+01	4.9612E+01	4.5686E+01	7.2915E+01	**5.4558E+00**	4.5796E+01	3.6159E+01	4.2471E+01
F22	Ave	2.3183E+03	2.6192E+03	2.4414E+03	2.3444E+03	2.3539E+03	2.3035E+03	2.3072E+03	2.3116E+03	**2.2979E+03**	2.3059E+03	2.3013E+03	2.3022E+03
	Std	1.2483E+01	7.3635E+02	3.0484E+02	6.2826E+01	3.0955E+01	2.8411E+00	2.7840E+00	1.9409E+01	2.0573E+01	2.2805E+00	1.3424E+01	**1.3491E+00**
F23	Ave	2.6542E+03	2.6975E+03	2.6697E+03	2.6523E+03	2.6559E+03	**2.6205E+03**	2.6368E+03	2.6408E+03	2.6290E+03	2.6264E+03	2.6213E+03	2.6213E+03
	Std	1.4934E+01	4.0552E+01	1.4664E+01	2.2704E+01	1.9938E+01	9.2054E+00	1.8799E+01	1.7355E+01	1.2620E+01	1.1938E+01	**6.7594E+00**	9.5027E+00
F24	Ave	2.7090E+03	2.8172E+03	2.7946E+03	2.7279E+03	2.7599E+03	2.7557E+03	2.7650E+03	2.7634E+03	**2.6520E+03**	2.7257E+03	2.7146E+03	2.7424E+03
	Std	1.1131E+02	7.9172E+01	1.6228E+01	1.0079E+02	7.6447E+01	**1.5077E+01**	7.3734E+01	7.6389E+01	1.1864E+02	9.1284E+01	9.0409E+01	6.7453E+01
F25	Ave	2.9434E+03	2.9408E+03	3.0062E+03	2.9890E+03	2.9651E+03	2.9382E+03	2.9293E+03	2.9316E+03	**2.9234E+03**	2.9315E+03	2.9253E+03	2.9310E+03
	Std	**2.0808E+01**	3.3741E+01	3.7362E+01	6.3902E+01	3.7266E+01	3.1846E+01	2.3613E+01	3.3858E+01	2.4861E+01	2.8869E+01	2.5235E+01	2.1687E+01
F26	Ave	3.0809E+03	3.7389E+03	3.2170E+03	3.6073E+03	3.2443E+03	3.2251E+03	3.2266E+03	3.0079E+03	2.9401E+03	3.1700E+03	2.9616E+03	**2.9174E+03**
	Std	1.7394E+02	6.1532E+02	2.4204E+02	3.8280E+02	2.5827E+02	4.3877E+02	2.1117E+02	2.8814E+02	**6.5869E+01**	4.8896E+02	2.1422E+02	6.7817E+01
F27	Ave	3.1072E+03	3.1787E+03	3.1195E+03	3.1367E+03	3.1108E+03	3.1043E+03	3.1175E+03	3.1212E+03	3.1015E+03	3.1066E+03	3.1016E+03	**3.0767E+03**
	Std	1.6984E+01	4.7406E+01	3.4700E+01	3.9809E+01	2.6291E+01	1.7562E+01	3.5192E+01	3.2625E+01	7.1334E+00	2.7693E+01	1.8105E+01	**3.0360E+00**
F28	Ave	3.3257E+03	3.4412E+03	3.4368E+03	3.4257E+03	3.3724E+03	3.3652E+03	3.3706E+03	3.3538E+03	**3.1828E+03**	3.3403E+03	3.3216E+03	3.2821E+03
	Std	9.3827E+01	2.2334E+02	1.4793E+02	**6.6363E+01**	1.2378E+02	1.6410E+02	8.9644E+01	2.2324E+02	7.5416E+01	1.1486E+02	1.2489E+02	1.8852E+01
F29	Ave	3.2539E+03	3.5006E+03	3.4062E+03	3.3708E+03	3.2801E+03	3.2197E+03	3.2690E+03	3.2605E+03	3.2334E+03	3.2435E+03	3.2175E+03	**3.2146E+03**
	Std	5.3181E+01	1.5820E+02	1.1856E+02	8.8628E+01	8.3117E+01	5.3756E+01	7.0128E+01	6.3361E+01	**4.0438E+01**	6.5762E+01	5.4879E+01	4.0533E+01
F30	Ave	6.5541E+05	1.2285E+06	5.1571E+06	9.1751E+05	6.9387E+05	4.1897E+05	4.1867E+05	3.1246E+06	2.4577E+05	2.8760E+05	2.9431E+05	**7.9801E+03**
	Std	7.8532E+05	1.4020E+06	4.7027E+06	8.8225E+05	8.0376E+05	5.9127E+05	7.3904E+05	1.0495E+07	4.6756E+05	5.0005E+05	4.8464E+05	**4.8162E+03**
(W|T|L)		(0|30|0)	(0|23|7)	(0|9|21)	(0|28|2)	(0|30|0)	(1|29|0)	(0|30|0)	(1|29|0)	(7|23|0)	(0|30|0)	(1|29|0)	(**20**|10|0)
Mean		7.73	9.30	11.50	10.40	8.27	4.47	6.93	5.50	3.77	4.20	3.90	**2.03**
Ranking		8	10	12	11	9	5	7	6	2	4	3	**1**

Significant values are in [bold].

**Table 5:** Experimental results of 12 algorithms on CEC 2017 (Dim = 30)

ID		PO	HLOA	LEA	HEOA	NRBO	MELGWO	HPHHO	PPSO	EWOA	SRIME	RIME	MIRIME
F1	Ave	9.5249E+09	1.3915E+08	2.9309E+10	1.8958E+10	1.6807E+10	1.8789E+09	2.3272E+09	3.1200E+07	1.7329E+07	3.6363E+06	4.7314E+06	**9.1459E+05**
	Std	4.2827E+09	6.5714E+07	8.3417E+09	6.4535E+09	4.4253E+09	1.7165E+09	1.3661E+09	1.2523E+07	2.1392E+07	1.6198E+06	1.7089E+06	**3.3217E+05**
F2	Ave	6.5680E+34	9.2433E+36	2.6364E+42	9.9362E+37	1.9029E+35	5.8973E+32	1.0000E+20	5.2106E+38	2.2984E+25	5.9920E+17	2.8724E+17	**1.2870E+17**
	Std	2.5226E+35	4.0705E+37	1.3850E+43	4.1751E+38	6.0570E+35	3.0418E+33	**0.0000E+00**	2.8539E+39	8.8969E+25	1.3373E+18	6.4529E+17	4.6414E+17
F3	Ave	5.4310E+04	3.6156E+04	3.0157E+05	7.2789E+04	5.4642E+04	4.3937E+04	4.7983E+04	3.0846E+04	1.1311E+05	5.6614E+04	5.2759E+04	**2.7662E+04**
	Std	9.2159E+03	1.0558E+04	9.1480E+04	**6.4572E+03**	9.6993E+03	8.7802E+03	9.6294E+03	9.6493E+03	2.1121E+04	1.9592E+04	1.6593E+04	7.2374E+03
F4	Ave	1.1163E+03	5.8238E+02	4.7675E+03	2.2844E+03	1.9889E+03	6.3551E+02	7.8568E+02	5.3454E+02	5.3818E+02	5.3608E+02	5.2472E+02	**4.8778E+02**
	Std	4.1213E+02	4.7832E+01	1.9477E+03	9.1932E+02	5.8875E+02	1.1846E+02	1.7722E+02	2.5105E+01	3.3718E+01	3.5412E+01	3.9179E+01	**1.8236E+01**
F5	Ave	7.8952E+02	8.1455E+02	9.5227E+02	8.6209E+02	8.4361E+02	6.8071E+02	7.8297E+02	7.4497E+02	6.5856E+02	6.8279E+02	6.2358E+02	**6.1924E+02**
	Std	4.8842E+01	4.5472E+01	4.9881E+01	3.4302E+01	4.3967E+01	4.2114E+01	3.5358E+01	4.9196E+01	4.0848E+01	5.6961E+01	**2.9968E+01**	3.5191E+01
F6	Ave	6.6719E+02	6.7538E+02	6.9528E+02	6.7783E+02	6.7520E+02	6.4203E+02	6.6016E+02	6.5925E+02	6.2983E+02	6.5378E+02	6.1340E+02	**6.0760E+02**
	Std	9.0993E+00	8.0308E+00	1.4810E+01	7.2467E+00	8.1973E+00	6.2530E+00	8.2081E+00	7.9556E+00	9.5163E+00	1.6371E+01	6.3983E+00	**5.7257E+00**
F7	Ave	1.2381E+03	1.3426E+03	1.8635E+03	1.3583E+03	1.2003E+03	9.9837E+02	1.2037E+03	1.1767E+03	9.8248E+02	9.6011E+02	**8.7428E+02**	8.7528E+02
	Std	6.5890E+01	5.9306E+01	2.7808E+02	7.7435E+01	7.2645E+01	5.4950E+01	7.4877E+01	9.2395E+01	4.9282E+01	4.2977E+01	**4.1702E+01**	4.7256E+01
F8	Ave	1.0422E+03	1.0331E+03	1.2196E+03	1.0888E+03	1.0768E+03	9.4192E+02	1.0120E+03	9.7536E+02	9.4989E+02	9.6975E+02	**9.1387E+02**	9.2068E+02
	Std	2.8816E+01	3.9978E+01	4.9078E+01	3.3758E+01	2.4987E+01	3.3852E+01	**2.3374E+01**	3.0188E+01	3.4351E+01	3.6843E+01	2.4992E+01	3.2174E+01
F9	Ave	7.2101E+03	7.1989E+03	2.4302E+04	8.8907E+03	7.4082E+03	3.8796E+03	6.7835E+03	5.4027E+03	4.4637E+03	4.9739E+03	**2.8442E+03**	3.4039E+03
	Std	1.2996E+03	1.3256E+03	5.1863E+03	1.0113E+03	1.0665E+03	**7.8227E+02**	9.1107E+02	1.3903E+03	2.2252E+03	3.3451E+03	1.4946E+03	2.9337E+03
F10	Ave	6.8787E+03	7.0894E+03	9.2079E+03	7.7082E+03	7.8550E+03	5.1545E+03	6.0753E+03	5.7445E+03	5.8930E+03	5.3220E+03	**4.5521E+03**	6.7220E+03
	Std	7.2460E+02	9.3298E+02	6.8447E+02	6.1873E+02	5.7675E+02	1.0123E+03	**5.6344E+02**	7.6683E+02	1.0247E+03	6.0751E+02	7.2698E+02	1.6737E+03
F11	Ave	2.0377E+03	1.4912E+03	1.7408E+04	5.4314E+03	2.7134E+03	1.4282E+03	1.6417E+03	1.3278E+03	1.4293E+03	1.3687E+03	1.3351E+03	**1.2428E+03**
	Std	5.3846E+02	1.6488E+02	6.9548E+03	1.6617E+03	7.3308E+02	2.6693E+02	2.1059E+02	5.5780E+01	9.9324E+01	6.1100E+01	5.7134E+01	**5.1957E+01**
F12	Ave	3.7291E+08	5.0473E+07	1.6295E+09	8.7415E+08	1.6893E+09	2.8991E+07	1.6834E+08	1.7145E+07	**2.1058E+06**	1.7163E+07	1.4969E+07	4.6126E+06
	Std	3.3590E+08	4.0583E+07	7.1307E+08	5.7431E+08	7.9244E+08	2.1048E+07	1.0161E+08	1.7381E+07	**1.6327E+06**	1.6490E+07	1.2153E+07	2.6977E+06
F13	Ave	3.4761E+07	1.1531E+06	7.5178E+08	1.6372E+08	4.2145E+08	1.0779E+05	2.4239E+06	1.9658E+05	2.5772E+04	1.3575E+05	1.8619E+05	**6.1610E+03**
	Std	1.0355E+08	3.4922E+06	7.1689E+08	6.3749E+08	5.3467E+08	5.5668E+04	2.5435E+06	8.0624E+05	1.7199E+04	8.1431E+04	3.0436E+05	**3.5961E+03**
F14	Ave	5.0211E+05	1.8177E+05	4.3251E+06	1.3363E+06	1.4834E+05	1.6711E+05	6.0675E+05	2.9990E+04	2.3467E+05	7.1613E+04	1.1234E+05	**2.7524E+04**
	Std	4.9546E+05	2.2339E+05	3.5007E+06	1.0752E+06	1.4918E+05	3.0384E+05	6.3998E+05	**3.9017E+04**	2.5927E+05	6.0454E+04	9.3134E+04	4.0348E+04
F15	Ave	6.5813E+05	3.6165E+04	9.4849E+07	3.0673E+06	9.9359E+05	2.4168E+04	1.0095E+05	1.3485E+04	7.3729E+03	1.6502E+04	1.7221E+04	**3.6256E+03**
	Std	1.3883E+06	2.9686E+04	9.2626E+07	3.2707E+06	1.4744E+06	1.8548E+04	1.1788E+05	1.0679E+04	8.1171E+03	1.0091E+04	1.2088E+04	**1.6456E+03**
F16	Ave	3.7145E+03	3.8718E+03	4.5601E+03	3.6602E+03	3.7423E+03	2.9370E+03	3.3795E+03	3.1662E+03	2.8412E+03	2.8691E+03	**2.7376E+03**	2.7559E+03
	Std	3.3187E+02	7.0117E+02	3.7608E+02	5.0676E+02	4.3292E+02	3.0665E+02	3.6067E+02	**2.9902E+02**	3.0080E+02	3.4384E+02	3.2186E+02	3.3761E+02
F17	Ave	2.6106E+03	3.0119E+03	3.2274E+03	2.6858E+03	2.6433E+03	2.3106E+03	2.3786E+03	2.4875E+03	2.4145E+03	2.3555E+03	2.2129E+03	**2.0762E+03**
	Std	2.4624E+02	3.7769E+02	2.9336E+02	3.5729E+02	3.4420E+02	2.1938E+02	1.8035E+02	2.8917E+02	2.3921E+02	1.9449E+02	1.7429E+02	**1.5214E+02**
F18	Ave	3.3416E+06	1.1206E+06	4.5415E+07	8.9718E+06	2.8986E+06	1.0405E+06	2.4981E+06	**2.3372E+05**	2.2629E+06	1.1246E+06	1.6191E+06	4.7004E+05
	Std	3.2883E+06	9.0023E+05	3.6798E+07	9.4267E+06	5.1695E+06	1.1100E+06	2.5454E+06	**2.2873E+05**	2.8662E+06	8.9631E+05	1.7076E+06	5.9730E+05
F19	Ave	4.0401E+06	6.1274E+05	2.2364E+08	1.0443E+07	1.1465E+07	7.2908E+04	1.0700E+06	3.6222E+04	1.1072E+04	1.6453E+04	1.4396E+04	**5.9701E+03**
	Std	2.7783E+06	1.0040E+06	2.0715E+08	7.8169E+06	1.0786E+07	1.7025E+05	1.7949E+06	7.7432E+04	1.0328E+04	1.1876E+04	1.0060E+04	**5.9492E+03**
F20	Ave	2.6994E+03	3.0170E+03	3.1933E+03	2.8715E+03	2.8590E+03	2.6090E+03	2.6403E+03	2.7727E+03	2.5924E+03	2.6667E+03	2.5585E+03	**2.4308E+03**
	Std	1.9808E+02	2.2139E+02	2.2468E+02	2.3284E+02	1.9852E+02	2.0788E+02	2.1046E+02	2.7727E+02	1.9000E+02	2.0606E+02	2.0633E+02	**1.7913E+02**
F21	Ave	2.5449E+03	2.6578E+03	2.6961E+03	2.6035E+03	2.5993E+03	2.4503E+03	2.5511E+03	2.5343E+03	2.4389E+03	2.4569E+03	2.4161E+03	**2.3927E+03**
	Std	4.9406E+01	7.2965E+01	5.0806E+01	3.9194E+01	4.3503E+01	3.7807E+01	4.9297E+01	6.5527E+01	3.9006E+01	5.9994E+01	2.4422E+01	**2.3997E+01**
F22	Ave	4.4064E+03	8.1459E+03	9.6191E+03	7.1495E+03	6.6635E+03	5.3948E+03	4.1848E+03	5.4873E+03	2.5291E+03	5.9023E+03	5.1341E+03	**2.3088E+03**
	Std	1.5980E+03	1.0812E+03	2.1070E+03	1.9756E+03	2.5349E+03	1.7736E+03	2.0447E+03	2.4872E+03	7.6673E+02	1.5831E+03	1.8123E+03	**2.2269E+00**
F23	Ave	3.0435E+03	3.3705E+03	3.0622E+03	3.1634E+03	3.0515E+03	2.8500E+03	2.9913E+03	3.1118E+03	2.8133E+03	2.8493E+03	2.7872E+03	**2.7751E+03**
	Std	7.1298E+01	2.3314E+02	5.6428E+01	9.8093E+01	5.4986E+01	4.4259E+01	8.3931E+01	1.0208E+02	5.2600E+01	5.4830E+01	**3.0358E+01**	4.3821E+01
F24	Ave	3.1452E+03	3.4970E+03	3.2450E+03	3.2569E+03	3.2019E+03	2.9743E+03	3.1765E+03	3.2251E+03	2.9751E+03	3.0156E+03	**2.9561E+03**	3.0152E+03
	Std	5.0430E+01	1.7991E+02	6.9889E+01	8.5180E+01	5.8818E+01	**3.5777E+01**	6.8330E+01	1.3124E+02	4.5992E+01	5.0761E+01	4.2823E+01	6.2633E+01
F25	Ave	3.1786E+03	2.9986E+03	5.5744E+03	3.3048E+03	3.4077E+03	3.0013E+03	3.0770E+03	2.9680E+03	2.9326E+03	2.9247E+03	2.9184E+03	**2.9074E+03**
	Std	8.8547E+01	3.5165E+01	1.0558E+03	1.4013E+02	1.6427E+02	4.4208E+01	5.5132E+01	3.6203E+01	3.2197E+01	2.5596E+01	2.9742E+01	**2.2340E+01**
F26	Ave	8.4380E+03	9.5014E+03	8.4303E+03	8.7908E+03	7.5131E+03	5.7327E+03	7.1137E+03	6.5997E+03	5.4320E+03	5.6344E+03	4.9418E+03	**4.6322E+03**
	Std	1.0456E+03	1.4694E+03	7.6799E+02	9.4391E+02	9.5258E+02	7.2226E+02	1.4410E+03	2.0211E+03	8.7867E+02	**6.3922E+02**	7.7527E+02	6.7377E+02
F27	Ave	3.3854E+03	3.6988E+03	3.4774E+03	3.5137E+03	3.4315E+03	3.3029E+03	3.3644E+03	3.3852E+03	3.2655E+03	3.2798E+03	3.2407E+03	**3.1901E+03**
	Std	9.0499E+01	3.9335E+02	9.2839E+01	1.3519E+02	9.5332E+01	4.9770E+01	8.4952E+01	9.3296E+01	2.5439E+01	3.9276E+01	1.9917E+01	**1.4417E+01**
F28	Ave	3.8176E+03	3.3416E+03	5.2679E+03	4.5363E+03	4.2366E+03	3.4697E+03	3.4697E+03	3.3166E+03	3.3029E+03	3.2892E+03	3.3074E+03	**3.2644E+03**
	Std	2.6575E+02	4.6201E+01	9.8256E+02	4.1479E+02	4.5382E+02	1.6350E+02	8.2780E+01	**3.0968E+01**	5.1423E+01	3.2503E+01	6.1172E+01	2.4893E+01
F29	Ave	4.8718E+03	6.1695E+03	5.8756E+03	5.4516E+03	5.0593E+03	4.3578E+03	4.4778E+03	4.6082E+03	4.0772E+03	4.2690E+03	4.0581E+03	**3.6364E+03**
	Std	3.4532E+02	1.0272E+03	6.8824E+02	3.9896E+02	3.5525E+02	3.0430E+02	2.6649E+02	2.9330E+02	**2.0520E+02**	2.5591E+02	2.4318E+02	1.7055E+02
F30	Ave	3.8784E+07	7.0499E+06	1.7396E+08	6.1564E+07	8.9430E+07	2.8876E+06	1.1535E+07	7.3584E+05	2.5114E+04	8.0481E+05	7.1675E+05	**1.7229E+04**
	Std	3.3950E+07	1.2043E+07	1.3046E+08	3.9323E+07	5.5285E+07	1.9683E+06	1.2234E+07	7.4276E+05	**2.1057E+04**	5.2608E+05	3.5534E+05	3.5313E+04
(W|T|L)		(0|30|0)	(0|25|5)	(0|6|24)	(0|30|0)	(0|29|1)	(0|30|0)	(0|30|0)	(1|29|0)	(1|29|0)	(0|30|0)	(5|25|0)	(**23**|7|0)
Mean		8.40	8.63	11.63	10.43	9.40	4.77	7.00	5.23	3.77	4.40	2.87	**1.47**
Ranking		8	9	12	11	10	5	7	6	3	4	2	**1**

Significant values are in [bold].

**Table 6:** Experimental results of 12 algorithms on CEC 2017 (Dim = 50)

ID		PO	HLOA	LEA	HEOA	NRBO	MELGWO	HPHHO	PPSO	EWOA	SRIME	RIME	MIRIME
F1	Ave	3.8815E+10	8.7306E+08	1.0210E+11	4.9015E+10	5.5857E+10	1.4662E+10	2.1014E+10	1.0570E+09	1.0345E+09	4.3159E+07	4.2208E+07	**1.3536E+07**
	Std	9.9995E+09	2.5144E+08	1.9356E+10	1.2816E+10	7.5441E+09	6.6278E+09	6.5445E+09	3.5035E+08	5.5527E+08	1.4432E+07	1.2456E+07	**4.1122E+06**
F2	Ave	6.3463E+64	4.2272E+71	1.7338E+79	7.4476E+70	2.4000E+65	5.5853E+53	**1.0000E+20**	3.3534E+60	5.9956E+55	7.6111E+44	2.6987E+42	1.7634E+36
	Std	2.2728E+65	2.3124E+72	9.1277E+79	3.4785E+71	1.0626E+66	2.7622E+54	**0.0000E+00**	1.5564E+61	3.2701E+56	4.1639E+45	1.4000E+43	8.0281E+36
F3	Ave	1.4282E+05	2.2630E+05	5.7242E+05	1.8880E+05	1.7169E+05	1.2478E+05	1.2585E+05	**1.1670E+05**	2.4097E+05	2.1327E+05	2.0963E+05	2.7867E+05
	Std	1.6149E+04	6.4696E+04	1.9476E+05	**1.2798E+04**	4.2616E+04	2.1606E+04	1.6827E+04	1.9119E+04	3.9601E+04	4.0182E+04	4.6567E+04	4.1688E+04
F4	Ave	5.4290E+03	9.4370E+02	2.0006E+04	1.0310E+04	9.0833E+03	2.1796E+03	2.6041E+03	8.8914E+02	8.1945E+02	6.8891E+02	6.9739E+02	**6.2303E+02**
	Std	1.9721E+03	1.4672E+02	4.7043E+03	4.1094E+03	2.5755E+03	8.7984E+02	7.7541E+02	1.2161E+02	8.6812E+01	6.8145E+01	7.2576E+01	**5.2124E+01**
F5	Ave	1.0540E+03	1.0082E+03	1.3958E+03	1.1525E+03	1.1254E+03	8.3627E+02	9.9744E+02	9.2629E+02	8.5062E+02	9.1065E+02	**7.5506E+02**	7.9762E+02
	Std	3.9816E+01	5.3658E+01	9.0382E+01	**3.3400E+01**	4.6168E+01	4.3468E+01	3.6887E+01	3.9575E+01	6.0650E+01	9.4639E+01	4.3504E+01	3.9950E+01
F6	Ave	6.8295E+02	6.8548E+02	7.2041E+02	6.9432E+02	6.9164E+02	6.5823E+02	6.7788E+02	6.7078E+02	6.4916E+02	6.6488E+02	6.3189E+02	**6.1842E+02**
	Std	7.6907E+00	7.4293E+00	1.2921E+01	6.0084E+00	6.9615E+00	8.5810E+00	**5.8277E+00**	6.7489E+00	1.0242E+01	1.1567E+01	6.8483E+00	7.4961E+00
F7	Ave	1.8250E+03	1.9057E+03	3.5579E+03	2.0032E+03	1.8091E+03	1.4366E+03	1.8144E+03	1.7596E+03	1.4892E+03	1.3184E+03	1.1335E+03	**1.1148E+03**
	Std	9.4086E+01	8.6509E+01	3.9804E+02	7.4750E+01	1.2333E+02	1.1064E+02	9.2660E+01	8.6820E+01	1.5115E+02	1.0655E+02	7.8730E+01	**7.0948E+01**
F8	Ave	1.3614E+03	1.3364E+03	1.6692E+03	1.4570E+03	1.4334E+03	1.1215E+03	1.2874E+03	1.2422E+03	1.1441E+03	1.1880E+03	1.0645E+03	**1.0169E+03**
	Std	4.0931E+01	5.3960E+01	7.8096E+01	5.3377E+01	4.8089E+01	4.4621E+01	**3.2657E+01**	5.4794E+01	7.3886E+01	7.3743E+01	4.7671E+01	4.3920E+01
F9	Ave	2.5723E+04	2.4047E+04	7.2478E+04	3.1058E+04	2.7270E+04	1.2468E+04	2.2542E+04	1.7637E+04	1.9956E+04	1.5676E+04	**1.1908E+04**	2.3356E+04
	Std	4.4719E+03	3.4947E+03	1.4557E+04	2.7700E+03	5.0967E+03	**1.5317E+03**	2.7177E+03	3.0475E+03	7.5945E+03	5.9116E+03	4.5908E+03	1.1789E+04
F10	Ave	1.2104E+04	1.2484E+04	1.6073E+04	1.3475E+04	1.3954E+04	8.7267E+03	1.0669E+04	9.6241E+03	1.1015E+04	8.6912E+03	**8.0546E+03**	9.5158E+03
	Std	1.0424E+03	1.0623E+03	7.7405E+02	**7.4591E+02**	8.1543E+02	1.0379E+03	9.4535E+02	9.5365E+02	1.6908E+03	9.1170E+02	1.0835E+03	2.8134E+03
F11	Ave	7.1156E+03	2.5193E+03	5.4842E+04	9.9643E+03	1.0357E+04	3.9038E+03	3.4685E+03	2.0144E+03	3.4057E+03	1.7625E+03	1.7372E+03	**1.4576E+03**
	Std	2.2105E+03	4.9434E+02	1.5397E+04	2.7435E+03	2.5800E+03	1.6428E+03	8.5336E+02	2.3953E+02	1.5876E+03	1.1928E+02	1.3330E+02	**7.3605E+01**
F12	Ave	5.0051E+09	4.0916E+08	1.7731E+10	1.5190E+10	1.4756E+10	1.6140E+09	1.5921E+09	3.0786E+08	4.3789E+07	2.1007E+08	1.4681E+08	**3.3643E+07**
	Std	2.6123E+09	2.6475E+08	5.5093E+09	8.7186E+09	7.3427E+09	2.3343E+09	8.4145E+08	4.2793E+08	2.6731E+07	1.1120E+08	6.7240E+07	**2.0814E+07**
F13	Ave	1.0475E+09	9.2717E+06	4.5120E+09	1.0111E+09	5.2699E+09	3.2033E+08	1.5032E+08	8.5713E+06	**3.4243E+04**	6.2798E+05	5.7446E+05	1.4660E+05
	Std	1.6865E+09	8.6773E+06	2.0872E+09	1.4179E+09	4.4649E+09	1.5801E+09	1.2217E+08	4.4875E+07	**2.1799E+04**	5.1871E+05	3.6531E+05	1.0071E+05
F14	Ave	5.2132E+06	1.9588E+06	2.6428E+07	5.4245E+06	2.6076E+06	1.1123E+06	1.8751E+06	7.2093E+05	1.2521E+06	5.4464E+05	6.7221E+05	**5.2892E+05**
	Std	3.9250E+06	1.6417E+06	1.9731E+07	5.2452E+06	2.3168E+06	1.1771E+06	1.2862E+06	1.4215E+06	9.7532E+05	4.1106E+05	4.3492E+05	**3.8321E+05**
F15	Ave	8.9812E+07	3.0869E+06	1.2524E+09	2.7142E+08	3.1205E+08	1.9569E+06	1.4448E+07	2.9790E+04	**9.9043E+03**	1.0877E+05	1.1451E+05	2.7211E+04
	Std	2.1783E+08	1.3465E+07	6.5908E+08	2.4474E+08	1.4788E+08	5.0427E+06	1.5682E+07	3.4293E+04	**6.7949E+03**	7.4538E+04	6.1683E+04	1.5141E+04
F16	Ave	5.4331E+03	5.8833E+03	6.9331E+03	6.1982E+03	6.1497E+03	4.0141E+03	4.7009E+03	4.4442E+03	3.5884E+03	4.1135E+03	3.7321E+03	**3.1612E+03**
	Std	8.1861E+02	5.9533E+02	7.2388E+02	1.1850E+03	7.5883E+02	6.1171E+02	5.1522E+02	6.3710E+02	4.8779E+02	4.9268E+02	4.3010E+02	**3.6015E+02**
F17	Ave	4.3614E+03	4.2733E+03	7.1573E+03	4.5605E+03	4.7239E+03	3.5520E+03	3.7800E+03	3.7268E+03	3.4786E+03	3.5872E+03	3.4449E+03	**3.0662E+03**
	Std	3.8049E+02	6.0426E+02	2.4644E+03	9.0992E+02	5.0127E+02	**3.0604E+02**	4.1727E+02	4.2667E+02	3.4466E+02	4.0730E+02	4.4617E+02	3.1743E+02
F18	Ave	1.9299E+07	3.1851E+06	1.4255E+08	5.1930E+07	2.6777E+07	7.2675E+06	1.0894E+07	**2.1179E+06**	5.6286E+06	4.9204E+06	6.2971E+06	2.5798E+06
	Std	1.6802E+07	1.7067E+06	8.8914E+07	2.6255E+07	2.7055E+07	7.5357E+06	1.1468E+07	2.2698E+06	4.9778E+06	3.7486E+06	3.8304E+06	**1.0942E+06**
F19	Ave	8.6175E+07	1.7839E+06	5.6110E+08	9.5615E+07	2.1882E+08	3.4732E+05	2.8107E+06	2.7064E+05	**1.7860E+04**	5.8916E+05	4.8241E+05	2.7922E+04
	Std	1.4309E+08	2.3749E+06	2.9499E+08	1.0016E+08	1.2825E+08	3.4659E+05	4.7863E+06	5.8870E+05	1.1595E+04	4.5533E+05	4.8672E+05	**9.6299E+03**
F20	Ave	3.6194E+03	4.1073E+03	4.6630E+03	3.8550E+03	3.9093E+03	3.2239E+03	3.3793E+03	3.6615E+03	3.3288E+03	3.5879E+03	3.3435E+03	**3.0831E+03**
	Std	3.5534E+02	4.1164E+02	3.0887E+02	**2.7282E+02**	3.3169E+02	2.7709E+02	2.8506E+02	3.6772E+02	3.1633E+02	3.8318E+02	2.7307E+02	2.9287E+02
F21	Ave	2.8720E+03	3.1247E+03	3.1848E+03	2.9901E+03	2.9688E+03	2.6601E+03	2.8597E+03	2.8220E+03	2.6334E+03	2.6931E+03	2.5643E+03	**2.5587E+03**
	Std	8.4445E+01	1.2690E+02	8.6989E+01	6.8036E+01	**5.3050E+01**	6.8032E+01	7.4440E+01	8.1950E+01	6.5249E+01	7.8101E+01	6.1744E+01	7.6712E+01
F22	Ave	1.3999E+04	1.4463E+04	1.7994E+04	1.4459E+04	1.5857E+04	1.0311E+04	1.2735E+04	1.1568E+04	1.2089E+04	1.0221E+04	**9.6516E+03**	1.1773E+04
	Std	9.6909E+02	1.2106E+03	8.2899E+02	9.6266E+02	**7.4795E+02**	9.0273E+02	1.2184E+03	9.5153E+02	1.8118E+03	8.6320E+02	1.6098E+03	5.2565E+03
F23	Ave	3.5984E+03	4.3075E+03	3.6859E+03	3.7969E+03	3.6102E+03	3.1643E+03	3.5198E+03	3.7312E+03	3.1549E+03	3.2060E+03	**3.0648E+03**	3.1136E+03
	Std	1.7598E+02	3.1600E+02	1.1733E+02	1.7046E+02	1.0830E+02	8.4903E+01	1.1225E+02	1.7803E+02	9.4901E+01	1.0867E+02	**6.9038E+01**	8.6681E+01
F24	Ave	3.6573E+03	4.4299E+03	3.8191E+03	3.8460E+03	3.7782E+03	3.2792E+03	3.7532E+03	4.1672E+03	3.2719E+03	3.3180E+03	**3.1935E+03**	3.3474E+03
	Std	1.1084E+02	2.8959E+02	1.0913E+02	1.2186E+02	1.4183E+02	**7.0800E+01**	1.4257E+02	3.6141E+02	8.6486E+01	1.1646E+02	9.1513E+01	1.3747E+02
F25	Ave	5.6734E+03	3.3958E+03	2.0095E+04	7.6023E+03	7.0731E+03	4.1252E+03	4.4758E+03	3.3596E+03	3.2585E+03	3.1493E+03	3.1459E+03	**3.0811E+03**
	Std	6.5445E+02	1.1824E+02	5.4424E+03	1.2124E+03	1.0079E+03	4.1323E+02	4.2764E+02	1.1098E+02	1.0481E+02	3.6659E+01	4.4464E+01	**3.2414E+01**
F26	Ave	1.2932E+04	1.3555E+04	1.4167E+04	1.4655E+04	1.3181E+04	1.0070E+04	1.2465E+04	1.2347E+04	7.9837E+03	8.0426E+03	7.1611E+03	**3.5319E+03**
	Std	1.4850E+03	1.3372E+03	1.4089E+03	1.4366E+03	1.8801E+03	1.5614E+03	1.6244E+03	1.8157E+03	**7.3003E+02**	1.3443E+03	7.6355E+02	1.5817E+03
F27	Ave	4.1540E+03	5.2195E+03	4.6312E+03	4.5733E+03	4.3584E+03	3.9241E+03	4.1578E+03	4.0145E+03	3.7126E+03	3.6782E+03	3.6265E+03	**3.2006E+03**
	Std	2.5750E+02	9.1871E+02	3.2422E+02	5.5966E+02	2.3222E+02	2.5474E+02	2.1703E+02	3.9894E+02	1.2886E+02	1.3528E+02	1.1984E+02	**3.3862E+00**
F28	Ave	6.0113E+03	3.7537E+03	1.0883E+04	7.7652E+03	7.1935E+03	4.9242E+03	5.3711E+03	3.8141E+03	3.7183E+03	3.4474E+03	3.4490E+03	**3.3255E+03**
	Std	5.1733E+02	2.0238E+02	9.2594E+02	7.3586E+02	7.2108E+02	3.7055E+02	3.5171E+02	2.2578E+02	2.4342E+02	6.3879E+01	**4.9497E+01**	4.1939E+01
F29	Ave	7.9884E+03	8.8825E+03	1.1887E+04	9.4141E+03	7.9986E+03	6.0958E+03	7.0024E+03	6.0493E+03	4.8598E+03	5.5686E+03	5.2215E+03	**4.4913E+03**
	Std	1.1992E+03	1.6308E+03	2.8872E+03	2.2853E+03	7.4946E+02	7.6621E+02	9.4007E+02	5.2142E+02	**3.1774E+02**	4.6756E+02	5.1224E+02	3.7434E+02
F30	Ave	3.8559E+08	1.4602E+08	1.4216E+09	6.0682E+08	8.8900E+08	9.8996E+07	1.9478E+08	3.5543E+07	3.4664E+06	5.5968E+07	6.3514E+07	**1.4062E+05**
	Std	2.5127E+08	5.9967E+07	4.8986E+08	7.4677E+08	6.0211E+08	4.7481E+07	6.2906E+07	2.4623E+07	1.2588E+06	2.6185E+07	2.4309E+07	**1.2108E+05**
(W|T|L)		(0|30|0)	(0|27|3)	(0|4|26)	(0|29|1)	(0|30|0)	(1|29|0)	(1|29|0)	(3|27|0)	(3|27|0)	(0|30|0)	(5|25|0)	(**17**|13|0)
Mean		8.40	8.20	11.73	10.27	9.70	4.53	6.93	5.10	3.87	4.03	3.00	**2.23**
Ranking		9	8	12	11	10	5	7	6	3	4	2	**1**

Significant values are in [bold].

#### Comparison with other competitive algorithms in CEC 2022

**Figure 11:**
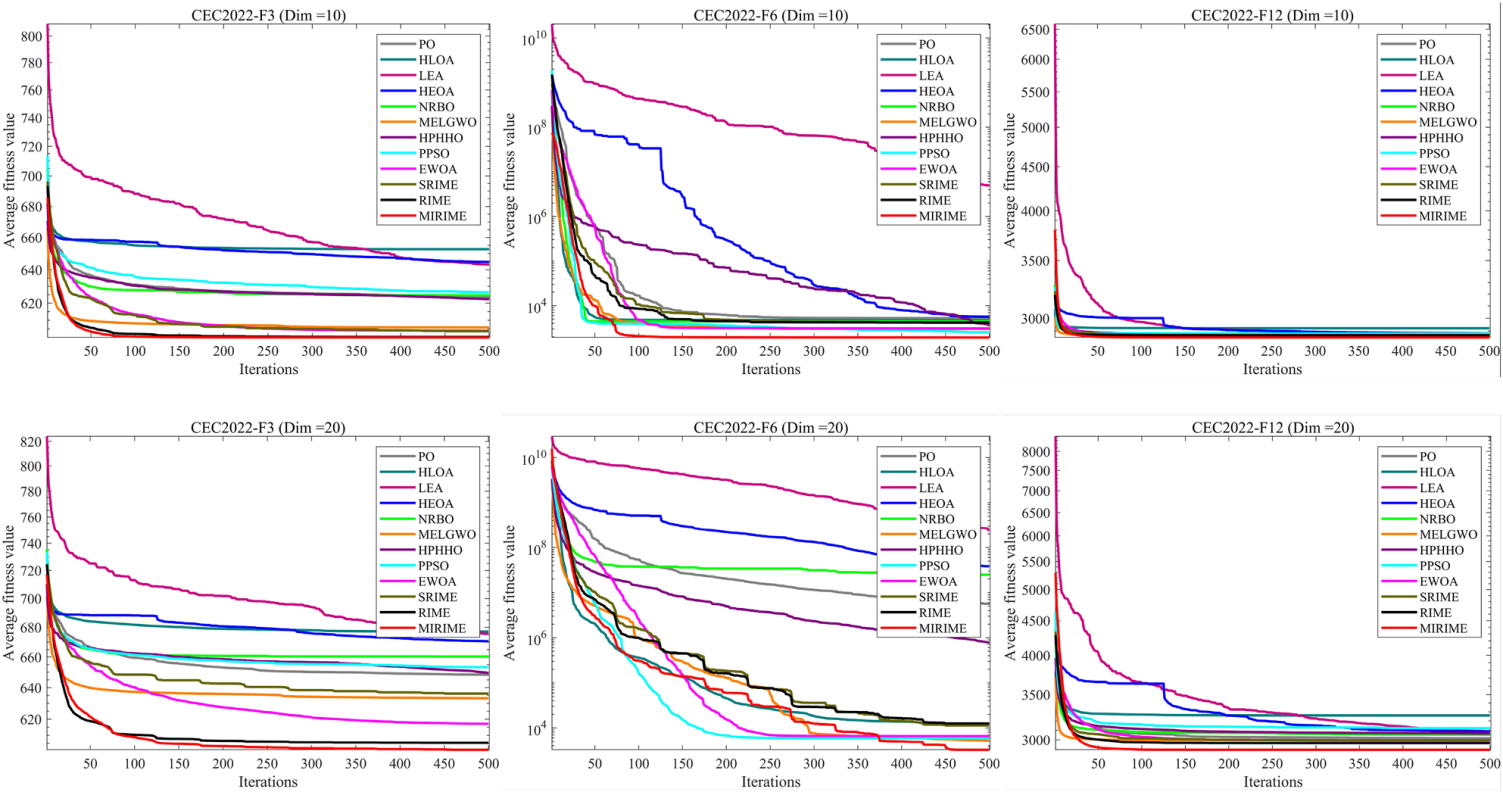
Comparison of convergence curves of 12 algorithms on CEC2022.

**Figure 12:**
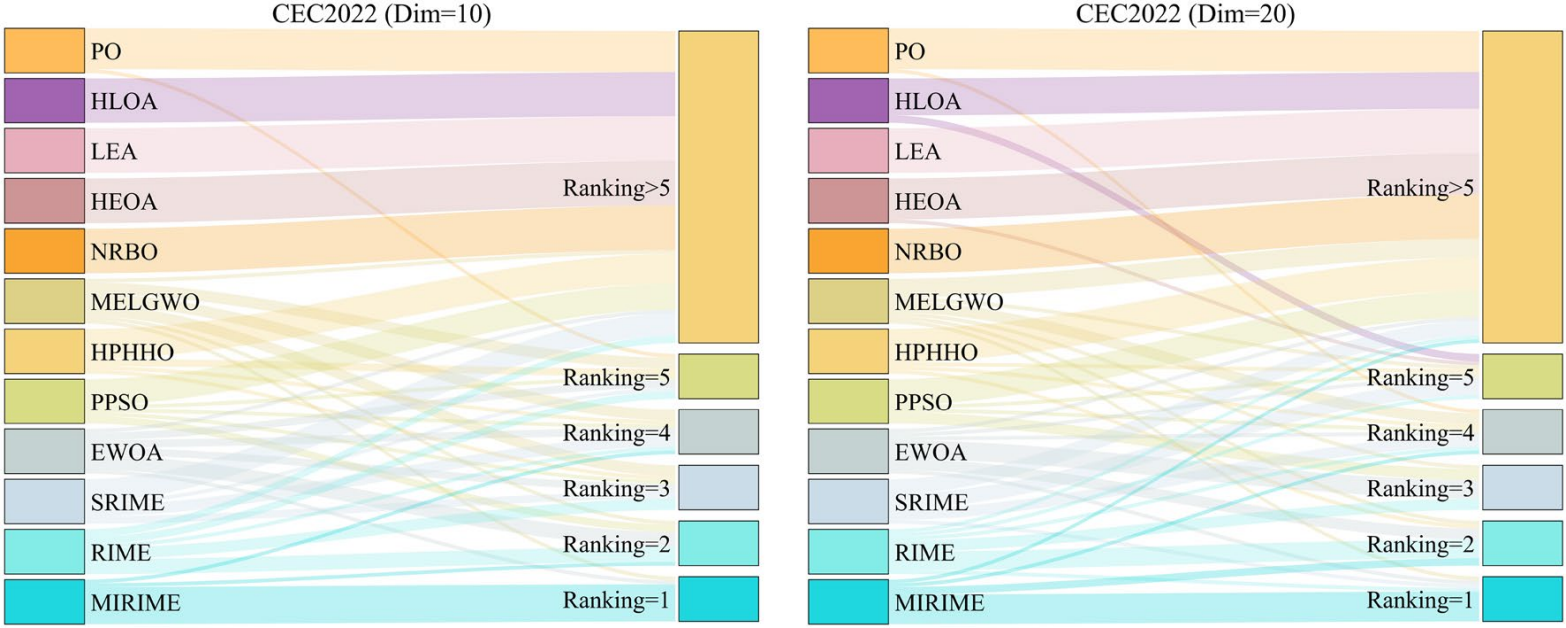
The ranking Sankey of different competitors on CEC2022.

**Figure 13:**
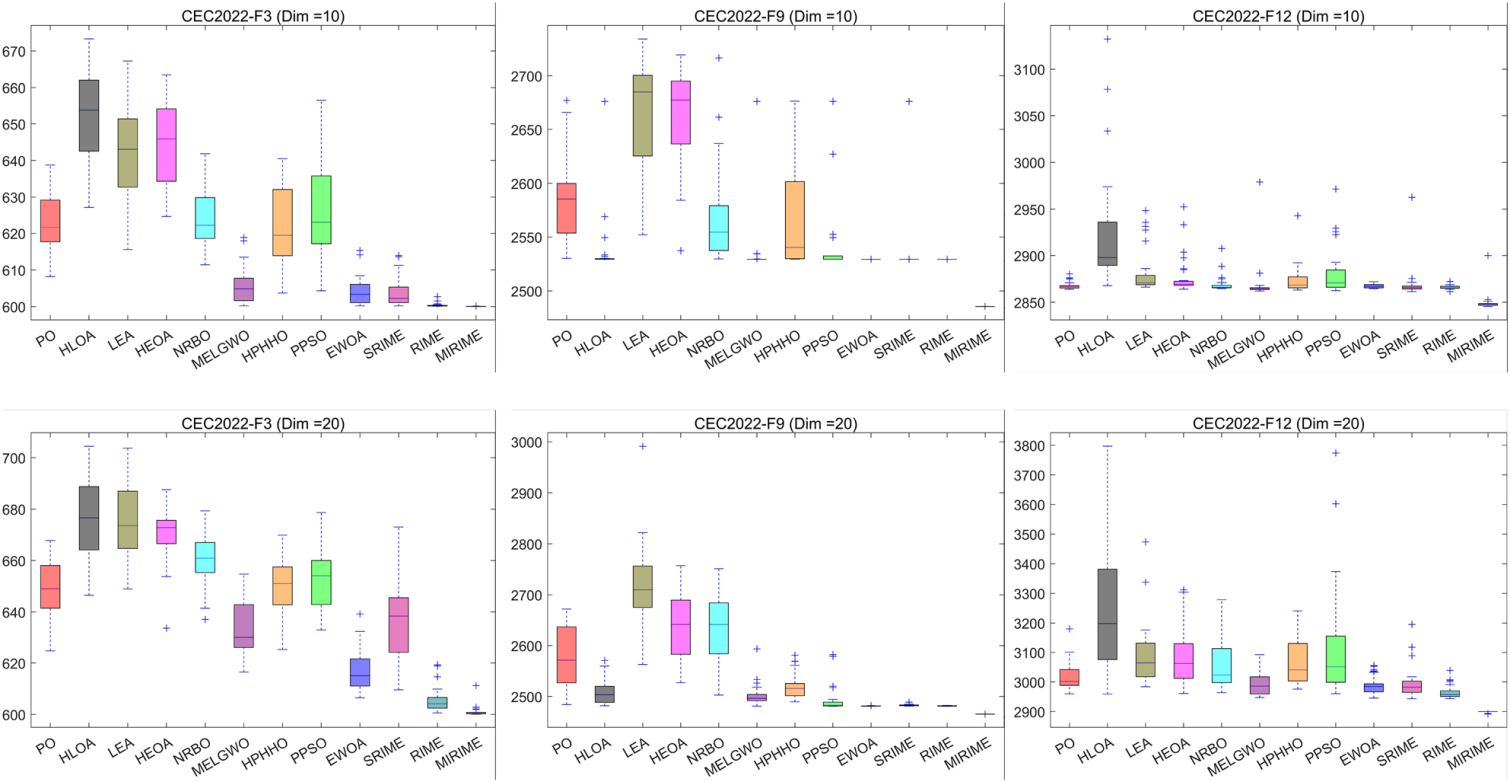
Boxplot analysis of competitor algorithms on the CEC2022 test suite.

We employed the CEC 2022 test suite to evaluate the performance of competing algorithms, with dimensions set at 10 and 20. The experimental results, detailed in Tables 7 and 8, reveal that MIRIME secures a significant number of wins (W) in all tested dimensions without any failures (L). To further investigate the convergence rate of the MIRIME algorithm, we conducted a comparative analysis with other algorithms. The experimental results are displayed in Fig. 11. Comparison of convergence curves of 12 algorithms on CEC2022. MIRIME's convergence curve gradually declines as the number of iterations increases, consistently maintaining a lower trajectory than that of other algorithms, which indicates a faster convergence rate and superior optimization capability relative to its competitors. Figure 12 illustrates the Friedman rankings of MIRIME and its comparative algorithms across 10 and 20 dimensions on the CEC2022 test set, using a Sankey diagram. In the diagram, it is clear that MIRIME predominantly targets the highest-ranked color region, while the other algorithms primarily occupy the lower-ranked color regions. This clearly demonstrates that MIRIME secures the highest number of first-place rankings in both dimensions. Figure 13 details the performance of 12 algorithms across two dimensions of the CEC2022 test set, depicted through box plots. The plots show that the MIRIME algorithm maintains a lower box position than its competitors, suggesting superior optimization values. Additionally, the narrowest bin width indicates that MIRIME achieves data consistency with minimal fluctuations and enhanced stability. Therefore, these factors collectively confirm that MIRIME delivers the best overall performance.

**Table 7:** Experimental results of 12 algorithms on CEC 2022 (Dim = 10)

ID		PO	HLOA	LEA	HEOA	NRBO	MELGWO	HPHHO	PPSO	EWOA	SRIME	RIME	MIRIME
F1	Ave	4.2299E+02	3.4856E+02	2.5423E+04	5.2538E+03	1.3500E+03	3.5193E+02	5.4378E+02	8.3873E+02	1.2416E+03	3.0095E+02	3.0091E+02	**3.0008E+02**
	Std	7.7120E+01	6.1248E+01	1.2119E+04	1.9174E+03	9.4226E+02	2.7334E+02	2.5690E+02	2.9469E+03	7.1784E+02	8.1505E−01	7.8480E−01	**9.8187E−02**
F2	Ave	4.2585E+02	4.2704E+02	4.6429E+02	4.4732E+02	4.5225E+02	4.1514E+02	4.2624E+02	4.1977E+02	4.1743E+02	**4.1080E+02**	4.1650E+02	4.1470E+02
	Std	2.7187E+01	3.3451E+01	4.3852E+01	3.6158E+01	2.6040E+01	2.1648E+01	3.0108E+01	2.9110E+01	2.6467E+01	**1.7310E+01**	2.7416E+01	2.4719E+01
F3	Ave	6.2323E+02	6.5266E+02	6.4319E+02	6.4469E+02	6.2464E+02	6.0581E+02	6.2232E+02	6.2645E+02	6.0390E+02	6.0365E+02	6.0036E+02	**6.0003E+02**
	Std	7.9029E+00	1.1753E+01	1.2842E+01	1.1308E+01	8.1893E+00	5.0730E+00	1.0734E+01	1.4164E+01	3.7917E+00	3.8914E+00	5.2198E−01	**3.2802E−02**
F4	Ave	8.2697E+02	8.4379E+02	8.6792E+02	8.3754E+02	8.3319E+02	**8.1900E+02**	8.2605E+02	8.3134E+02	8.2424E+02	8.2985E+02	8.2849E+02	8.2690E+02
	Std	9.5154E+00	1.5948E+01	1.6334E+01	6.7862E+00	8.7150E+00	7.0177E+00	6.8992E+00	9.1739E+00	1.0081E+01	1.1235E+01	1.1001E+01	**6.2138E+00**
F5	Ave	1.1472E+03	1.3866E+03	1.8928E+03	1.4536E+03	1.0947E+03	9.4694E+02	1.2390E+03	1.1619E+03	9.6352E+02	9.0529E+02	9.0104E+02	**9.0030E+02**
	Std	1.9347E+02	1.5342E+02	7.0936E+02	1.8391E+02	1.0698E+02	5.8976E+01	1.8701E+02	1.3868E+02	5.1904E+01	1.2555E+01	1.5561E+00	**5.7034E−01**
F6	Ave	5.2674E+03	4.6771E+03	4.9753E+06	5.5820E+03	4.4317E+03	3.0108E+03	3.5593E+03	2.5349E+03	3.0948E+03	4.2310E+03	4.1710E+03	**1.9393E+03**
	Std	2.2516E+03	2.2748E+03	4.6166E+06	2.7900E+03	2.1260E+03	1.3692E+03	2.1707E+03	1.5991E+03	1.2149E+03	2.0262E+03	2.2853E+03	**2.5332E+02**
F7	Ave	2.0571E+03	2.1240E+03	2.0897E+03	2.0996E+03	2.0606E+03	2.0375E+03	2.0332E+03	2.0445E+03	2.0279E+03	2.0390E+03	2.0214E+03	**2.0103E+03**
	Std	1.8246E+01	4.7328E+01	3.4360E+01	3.4393E+01	1.6687E+01	2.4247E+01	1.2104E+01	2.6984E+01	1.3227E+01	3.2883E+01	2.0626E+01	**9.5679E+00**
F8	Ave	2.2299E+03	2.3143E+03	2.2590E+03	2.2308E+03	2.2466E+03	2.2237E+03	2.2276E+03	2.2340E+03	2.2210E+03	2.2338E+03	**2.2171E+03**	2.2179E+03
	Std	3.2356E+00	9.2855E+01	3.0936E+01	7.2280E+00	4.2288E+01	4.7995E+00	**2.9216E+00**	3.0621E+01	4.5713E+00	3.0188E+01	7.6367E+00	6.9447E+00
F9	Ave	2.5854E+03	2.5414E+03	2.6652E+03	2.6631E+03	2.5687E+03	2.5442E+03	2.5778E+03	2.5405E+03	2.5293E+03	2.5391E+03	2.5293E+03	**2.4855E+03**
	Std	3.6721E+01	3.7492E+01	4.9171E+01	4.4300E+01	4.4117E+01	4.4779E+01	5.8472E+01	3.1705E+01	5.2878E−03	3.7279E+01	2.6968E−03	**1.5216E−03**
F10	Ave	2.5192E+03	2.8084E+03	2.5725E+03	2.6406E+03	2.5558E+03	2.5555E+03	2.5347E+03	2.5507E+03	**2.5008E+03**	2.5872E+03	2.5352E+03	2.5349E+03
	Std	4.4364E+01	5.1968E+02	8.3952E+01	1.1722E+01	6.8112E+01	6.0217E+01	5.7458E+01	7.4830E+01	**1.5126E−01**	1.0094E+02	5.8440E+01	5.3753E+01
F11	Ave	2.7662E+03	2.8418E+03	2.9619E+03	2.7646E+03	2.9242E+03	2.7192E+03	2.6968E+03	2.7062E+03	2.6553E+03	2.7270E+03	2.6831E+03	**2.6402E+03**
	Std	1.4447E+02	2.1756E+02	2.4820E+02	1.0603E+02	2.2905E+02	1.8197E+02	**7.2258E+01**	1.4800E+02	7.3850E+01	1.5539E+02	1.5115E+02	8.7540E+01
F12	Ave	2.8677E+03	2.9211E+03	2.8821E+03	2.8769E+03	2.8691E+03	2.8689E+03	2.8741E+03	2.8806E+03	2.8674E+03	2.8693E+03	2.8660E+03	**2.8497E+03**
	Std	4.0582E+00	6.2291E+01	2.3507E+01	2.0222E+01	8.6867E+00	2.1042E+01	1.5732E+01	2.5048E+01	**2.1324E+00**	1.7826E+01	2.2698E+00	9.6229E+00
(W|T|L)		(0|12|0)	(0|9|3)	(0|4|8)	(0|11|1)	(0|12|0)	(1|11|0)	(0|12|0)	(0|12|0)	(1|11|0)	(0|12|0)	(0|12|0)	(**10**|2|0)
Mean		7.75	9.67	11.58	10.42	8.75	3.75	6.42	5.92	3.58	5.17	3.67	**1.33**
Ranking		8	10	12	11	9	4	7	6	2	5	3	**1**

Significant values are in [bold].

**Table 8:** Experimental results of 12 algorithms on CEC 2022 (Dim = 20)

ID		PO	HLOA	LEA	HEOA	NRBO	MELGWO	HPHHO	PPSO	EWOA	SRIME	RIME	MIRIME
F1	Ave	1.3263E+04	8.6922E+03	8.7406E+04	2.9661E+04	1.5533E+04	6.5064E+03	7.9779E+03	4.5006E+03	2.2289E+04	**1.2829E+03**	1.4779E+03	1.2048E+04
	Std	2.9055E+03	8.2058E+03	2.4908E+04	7.2504E+03	4.9036E+03	2.7058E+03	3.5520E+03	4.5883E+03	5.3910E+03	**5.0098E+02**	8.4493E+02	6.8236E+03
F2	Ave	6.5125E+02	4.9910E+02	9.9331E+02	7.9096E+02	7.4205E+02	5.0383E+02	5.4345E+02	4.7357E+02	4.6543E+02	4.8294E+02	4.5698E+02	**4.3590E+02**
	Std	1.0043E+02	5.3172E+01	2.6688E+02	1.3208E+02	1.4494E+02	5.7367E+01	5.0271E+01	2.4158E+01	3.2820E+01	5.1884E+01	**2.3781E+01**	2.5988E+01
F3	Ave	6.4837E+02	6.7711E+02	6.7561E+02	6.7055E+02	6.6030E+02	6.3315E+02	6.4954E+02	6.5328E+02	6.1712E+02	6.3616E+02	6.0538E+02	**6.0105E+02**
	Std	1.0760E+01	1.4249E+01	1.5176E+01	1.0432E+01	1.0403E+01	1.0270E+01	1.0347E+01	1.1833E+01	7.9799E+00	1.6255E+01	4.7147E+00	**2.0796E+00**
F4	Ave	9.0713E+02	9.1896E+02	1.0063E+03	9.3848E+02	9.3199E+02	**8.6391E+02**	8.9458E+02	8.9256E+02	8.6614E+02	8.9387E+02	8.6726E+02	8.8033E+02
	Std	2.1703E+01	3.6811E+01	2.8150E+01	2.2015E+01	1.9710E+01	**1.4665E+01**	1.5317E+01	2.3384E+01	1.9444E+01	3.7884E+01	2.2869E+01	1.8659E+01
F5	Ave	2.5013E+03	2.7114E+03	7.7054E+03	3.1983E+03	2.4360E+03	1.5687E+03	2.6599E+03	2.2295E+03	1.9671E+03	1.7943E+03	**1.2546E+03**	1.3064E+03
	Std	2.8057E+02	4.0760E+02	1.9589E+03	3.2673E+02	5.0985E+02	2.9938E+02	**2.1177E+02**	3.0988E+02	6.9727E+02	9.3040E+02	5.7639E+02	8.0442E+02
F6	Ave	5.6490E+06	1.1737E+04	2.3857E+08	3.8146E+07	2.4900E+07	5.1777E+03	7.7945E+05	5.8878E+03	6.5662E+03	1.1335E+04	1.2564E+04	**3.2301E+03**
	Std	8.9898E+06	1.8785E+04	2.1408E+08	4.9453E+07	2.3634E+07	3.7153E+03	1.2638E+06	5.3007E+03	4.8375E+03	6.6773E+03	6.1328E+03	**1.8750E+03**
F7	Ave	2.1650E+03	2.3228E+03	2.2894E+03	2.2292E+03	2.1762E+03	2.1281E+03	2.1278E+03	2.1472E+03	2.0849E+03	2.1618E+03	2.0777E+03	**2.0725E+03**
	Std	3.6829E+01	8.8537E+01	7.8927E+01	6.9540E+01	5.0367E+01	6.1210E+01	3.9039E+01	6.1451E+01	2.6572E+01	7.8035E+01	**2.6111E+01**	4.3901E+01
F8	Ave	2.2849E+03	2.5331E+03	2.5175E+03	2.2503E+03	2.3079E+03	2.2800E+03	2.2511E+03	2.2827E+03	2.2504E+03	2.2889E+03	2.2597E+03	**2.2495E+03**
	Std	5.9512E+01	1.7776E+02	1.1336E+02	**2.3784E+01**	7.6252E+01	7.0158E+01	3.5614E+01	6.7051E+01	4.4980E+01	6.8117E+01	6.0934E+01	3.8380E+01
F9	Ave	2.5760E+03	2.5076E+03	2.7182E+03	2.6386E+03	2.6356E+03	2.5010E+03	2.5190E+03	2.4926E+03	2.4813E+03	2.4830E+03	2.4814E+03	**2.4654E+03**
	Std	5.8566E+01	2.3898E+01	7.8905E+01	6.2337E+01	6.8675E+01	2.1421E+01	2.3269E+01	2.5645E+01	3.3402E−01	1.8815E+00	4.8948E−01	**6.1520E−02**
F10	Ave	2.8463E+03	5.2564E+03	5.4070E+03	5.0748E+03	5.2889E+03	3.6443E+03	2.5079E+03	4.0106E+03	2.5125E+03	3.9204E+03	2.8723E+03	**2.5007E+03**
	Std	8.6859E+02	9.1459E+02	1.8962E+03	1.0247E+03	1.4631E+03	8.0858E+02	3.5238E+01	9.4471E+02	4.3013E+01	5.5113E+02	2.9153E+02	**1.2531E−01**
F11	Ave	4.5578E+03	3.0810E+03	4.9553E+03	6.7328E+03	4.9618E+03	3.1128E+03	3.4409E+03	2.9629E+03	2.9739E+03	3.1489E+03	2.9857E+03	**2.9330E+03**
	Std	7.5697E+02	1.4232E+02	7.1324E+02	1.0590E+03	8.2719E+02	2.5266E+02	1.8716E+02	2.1017E+02	1.1365E+02	8.8170E+02	1.1527E+02	**4.1864E+01**
F12	Ave	3.0171E+03	3.2601E+03	3.0887E+03	3.0851E+03	3.0545E+03	2.9943E+03	3.0656E+03	3.1210E+03	2.9895E+03	2.9949E+03	2.9658E+03	**2.8987E+03**
	Std	4.8435E+01	2.1557E+02	1.0330E+02	9.3465E+01	8.3240E+01	3.7775E+01	7.7165E+01	1.9032E+02	3.0055E+01	5.2890E+01	2.2734E+01	**2.9224E+00**
(W|T|L)		(0|12|0)	(0|9|3)	(0|4|8)	(0|11|1)	(0|12|0)	(1|11|0)	(0|12|0)	(0|12|0)	(1|11|0)	(1|11|0)	(1|11|0)	(**8**|4|0)
Mean		7.75	8.92	11.67	10.17	9.25	4.50	6.67	5.75	3.42	5.00	3.00	**1.92**
Ranking		8	9	12	11	10	4	7	6	3	5	2	**1**

Significant values are in [bold].

### Statistical analysis

In this section, we will statistically analyze the performance difference between MIRIME algorithm and other algorithms by analyzing the experimental data through Wilcoxon test and Friedman test.

#### Wilcoxon rank sum test

We employed the Wilcoxon rank-sum test^49^ to compare the MIRIME algorithm with other algorithms, and the results are detailed in Tables 9, 10, 11, 12, 13 and 14. When the p-value is lower than 0.05, there is a significant difference between MIRIME and other algorithms. Conversely, if there is no significant difference, such results are highlighted in bold in the table. On the CEC 2017 and CEC 2022 test set, as the problem dimension increases, the number of bold entries decreases, indicating that the difference between MIRIME and other algorithms becomes more significant. The notation “ + / = /-” is used to indicate whether MIRIME's performance is superior, equivalent, or inferior to its competitors. The data from Table 14 indicates that as the dimension increases, the performance gap between MIRIME and other algorithms gradually widens. Based on the analysis presented earlier in this paper, MIRIME significantly outperforms other competitors in comprehensive performance.

**Table 9:** P-value of 12 algorithms on CEC 2017 (Dim = 10)

ID	PO	HLOA	LEA	HEOA	NRBO	MELGWO	HPHHO	PPSO	EWOA	SRIME	RIME
F1	3.0199E−11	2.0338E−09	3.0199E−11	3.0199E−11	3.0199E−11	4.3584E−02	3.0199E−11	**2.7719E**−**01**	**9.5873E**−**01**	5.5727E−10	1.0937E−10
F2	1.7203E−12	1.7203E−12	1.7203E−12	1.7203E−12	1.7203E−12	3.6928E−11	1.7203E−12	1.9256E−12	9.1261E−12	9.2896E−10	1.5078E−10
F3	3.0199E−11	3.0199E−11	3.0199E−11	3.0199E−11	3.0199E−11	2.5974E−05	3.0199E−11	4.4440E−07	3.0199E−11	8.1527E−11	4.0772E−11
F4	1.0407E−04	**1.4532E**−**01**	7.3891E−11	3.4971E−09	7.3803E−10	**2.0095E**−**01**	1.6955E−02	**1.2235E**−**01**	**1.6238E**−**01**	**2.3399E**−**01**	**4.1191E**−**01**
F5	1.6947E−09	4.0772E−11	3.0199E−11	3.6897E−11	1.2057E−10	1.8575E−03	1.2023E−08	2.1947E−08	1.6351E−05	6.5277E−08	**4.2896E**−**01**
F6	3.0199E−11	3.0199E−11	3.0199E−11	3.0199E−11	3.0199E−11	3.0199E−11	3.0199E−11	3.0199E−11	3.0199E−11	3.0199E−11	3.0199E−11
F7	3.0199E−11	3.0199E−11	3.0199E−11	3.0199E−11	3.0199E−11	4.6856E−08	3.0199E−11	1.0937E−10	1.5581E−08	1.8731E−07	**9.0490E−02**
F8	1.2023E−08	3.6459E−08	3.0199E−11	4.9752E−11	2.2273E−09	**4.5530E−01**	2.1947E−08	9.8329E−08	1.0035E−03	7.2951E−04	**6.8432E−01**
F9	3.0199E−11	3.0199E−11	3.0199E−11	3.0199E−11	3.0199E−11	4.9752E−11	3.0199E−11	3.0199E−11	5.5727E−10	4.5726E−09	5.0922E−08
F10	1.1023E−08	1.3289E−10	6.0658E−11	4.9752E−11	4.1997E−10	7.6588E−05	8.3520E−08	8.8910E−10	1.0277E−06	5.9706E−05	**8.2357E−02**
F11	2.1544E−10	4.5043E−11	3.0199E−11	3.0199E−11	3.0199E−11	4.9752E−11	3.0199E−11	3.4742E−10	6.7650E−05	6.5277E−08	1.3017E−03
F12	3.0199E−11	2.8716E−10	3.0199E−11	3.0199E−11	4.6159E−10	8.9934E−11	7.6950E−08	4.9426E−05	1.5969E−03	8.1527E−11	3.8053E−07
F13	**8.7710E−02**	**1.2597E−01**	3.6897E−11	**5.0120E−02**	6.6689E−03	**2.5805E−01**	**7.5059E−01**	3.0939E−06	**8.6499E−01**	**1.8090E−01**	**2.2257E−01**
F14	1.3111E−08	5.5329E−08	2.3715E−10	3.0811E−08	9.8329E−08	1.0666E−07	8.3520E−08	5.8587E−06	7.0881E−08	2.1540E−06	3.6459E−08
F15	7.1186E−09	2.1947E−08	4.5043E−11	3.0199E−11	1.5964E−07	2.1947E−08	1.1567E−07	1.7290E−06	2.6015E−08	3.1573E−05	3.8249E−09
F16	6.7220E−10	4.8413E−02	6.9724E−03	2.6077E−02	**7.2446E−02**	1.2541E−07	3.9881E−04	3.0939E−06	2.0338E−09	1.3111E−08	6.6955E−11
F17	6.0459E−07	4.6159E−10	3.0199E−11	1.7769E−10	2.7829E−07	1.9527E−03	7.9590E−03	2.2539E−04	**9.4696E−01**	2.5974E−05	**9.1171E−01**
F18	1.6947E−09	2.5721E−07	3.0199E−11	5.0723E−10	4.1178E−06	5.0922E−08	2.0283E−07	2.1265E−04	8.3520E−08	9.5332E−07	1.7290E−06
F19	7.3891E−11	9.7555E−10	3.0199E−11	3.0199E−11	2.6695E−09	4.1997E−10	1.6132E−10	5.1857E−07	6.0658E−11	2.6806E−04	2.3715E−10
F20	3.3874E−02	2.6695E−09	5.5999E−07	1.2870E−09	3.1830E−03	**1.9579E−01**	1.9112E−02	**2.7719E−01**	1.9112E−02	**3.9527E−01**	**8.5000E−02**
F21	2.4157E−02	4.3106E−08	2.3800E−03	1.3853E−06	6.3560E−05	**8.7663E−01**	8.5641E−04	**3.9527E−01**	1.1077E−06	2.4157E−02	**5.3951E−01**
F22	4.0772E−11	1.0105E−08	3.0199E−11	9.5139E−06	5.5727E−10	2.2360E−02	4.1997E−10	1.5581E−08	**8.7710E−02**	3.8249E−09	1.0407E−04
F23	3.8202E−10	1.2057E−10	3.6897E−11	4.9980E−09	2.6099E−10	**7.7312E−01**	3.7704E−04	1.4298E−05	1.1228E−02	**8.7710E−02**	**7.3940E−01**
F24	**2.5188E−01**	4.3106E−08	4.1825E−09	**2.9727E−01**	6.7650E−05	**4.9178E−01**	1.6813E−04	1.8575E−03	4.2175E−04	**6.8432E−01**	4.0595E−02
F25	8.3146E−03	1.8575E−03	2.3715E−10	1.4298E−05	1.3250E−04	9.8834E−03	**5.3685E−02**	**1.8090E−01**	**6.4142E−01**	**1.6687E−01**	**6.2040E−01**
F26	2.6784E−06	1.1737E−09	1.3289E−10	4.9752E−11	1.7769E−10	5.4620E−06	7.3803E−10	8.1200E−04	**2.1702E−01**	8.8829E−06	1.1674E−05
F27	3.0199E−11	3.0199E−11	3.0199E−11	3.0199E−11	3.0199E−11	3.0199E−11	3.0199E−11	3.0199E−11	3.0199E−11	3.0199E−11	3.0199E−11
F28	**1.3345E−01**	1.4423E−03	2.1265E−04	5.0723E−10	2.8389E−04	2.1506E−02	2.8389E−04	**7.0092E−02**	2.3768E−07	3.5638E−04	2.5101E−02
F29	5.5699E−03	8.1014E−10	6.7220E−10	2.9215E−09	1.6798E−03	**9.0000E−01**	1.4423E−03	6.0971E−03	**7.2446E−02**	**7.9782E−02**	**9.8231E−01**
F30	2.3715E−10	6.6955E−11	3.0199E−11	3.3301E−11	7.1186E−09	7.7725E−09	3.0199E−11	3.5905E−05	2.3715E−10	1.4110E−09	6.1210E−10

Significant values are in [bold].

**Table 10:** P-value of 12 algorithms on CEC 2017 (Dim = 30)

ID	PO	HLOA	LEA	HEOA	NRBO	MELGWO	HPHHO	PPSO	EWOA	SRIME	RIME
F1	3.0199E−11	3.0199E−11	3.0199E−11	3.0199E−11	3.0199E−11	3.0199E−11	3.0199E−11	3.0199E−11	3.1589E−10	3.3384E−11	3.0199E−11
F2	3.0199E−11	3.0199E−11	3.0199E−11	3.0199E−11	3.0199E−11	3.0199E−11	1.2118E−12	3.0199E−11	3.0199E−11	5.9706E−05	1.7836E−04
F3	1.0937E−10	1.2362E−03	3.0199E−11	3.0199E−11	6.0658E−11	4.6856E−08	1.5465E−09	**2.3985E−01**	3.0199E−11	7.1186E−09	9.2603E−09
F4	3.0199E−11	1.3289E−10	3.0199E−11	3.0199E−11	3.0199E−11	8.9934E−11	3.0199E−11	8.4848E−09	1.6980E−08	1.4294E−08	7.5991E−07
F5	4.5043E−11	3.6897E−11	3.0199E−11	3.0199E−11	3.0199E−11	1.1077E−06	3.0199E−11	1.6132E−10	2.2539E−04	1.0188E−05	**5.9969E−01**
F6	3.0199E−11	3.0199E−11	3.0199E−11	3.0199E−11	3.0199E−11	3.0199E−11	3.0199E−11	3.0199E−11	2.1544E−10	3.0199E−11	1.6813E−04
F7	3.0199E−11	3.0199E−11	3.0199E−11	3.0199E−11	3.0199E−11	1.1737E−09	3.0199E−11	3.6897E−11	4.1825E−09	7.0881E−08	**9.8231E−01**
F8	8.9934E−11	2.1544E−10	3.0199E−11	3.6897E−11	3.3384E−11	5.5699E−03	4.6159E−10	3.3520E−08	4.9818E−04	1.1937E−06	**4.7335E−01**
F9	2.1540E−06	1.0277E−06	3.0199E−11	5.0922E−08	7.0430E−07	3.0339E−03	3.3242E−06	5.2650E−05	9.4683E−03	3.5012E−03	**7.8446E−01**
F10	**6.7350E−01**	**8.0727E−01**	3.4971E−09	**7.2446E−02**	2.3243E−02	1.5178E−03	4.3584E−02	1.3832E−02	3.7782E−02	1.5969E−03	4.4205E−06
F11	3.3384E−11	8.1527E−11	3.0199E−11	3.0199E−11	3.0199E−11	4.1825E−09	3.3384E−11	5.5999E−07	3.8202E−10	5.4617E−09	6.0459E−07
F12	3.0199E−11	3.8202E−10	3.0199E−11	3.0199E−11	3.0199E−11	5.5999E−07	3.0199E−11	1.9963E−05	9.2113E−05	1.9963E−05	3.5923E−05
F13	3.0199E−11	3.0199E−11	3.0199E−11	3.0199E−11	3.0199E−11	3.6897E−11	3.0199E−11	7.3891E−11	3.8053E−07	3.0199E−11	3.0199E−11
F14	1.2870E−09	2.5974E−05	3.0199E−11	7.3891E−11	4.9426E−05	3.5638E−04	2.2273E−09	**7.0617E−01**	1.4733E−07	2.9590E−05	1.8500E−08
F15	3.0199E−11	4.0772E−11	3.0199E−11	3.0199E−11	3.0199E−11	5.4941E−11	3.0199E−11	2.3168E−06	**6.5671E−02**	1.9568E−10	6.1210E−10
F16	2.8716E−10	3.4971E−09	3.0199E−11	1.1737E−09	9.7555E−10	4.5146E−02	6.5277E−08	2.5974E−05	**2.7719E−01**	**2.5805E−01**	**7.6183E−01**
F17	4.6159E−10	3.0199E−11	3.0199E−11	9.2603E−09	8.8910E−10	5.9706E−05	1.0666E−07	1.3594E−07	6.5261E−07	7.0430E−07	9.4683E−03
F18	3.3520E−08	4.7138E−04	3.0199E−11	7.1186E−09	1.0188E−05	1.5969E−03	3.2555E−07	**9.0490E−02**	6.2828E−06	4.7138E−04	1.4067E−04
F19	3.0199E−11	2.0338E−09	3.0199E−11	3.0199E−11	3.0199E−11	3.3520E−08	9.9186E−11	1.4067E−04	2.5101E−02	2.0023E−06	1.7479E−05
F20	3.8349E−06	3.4742E−10	3.6897E−11	2.1947E−08	3.1967E−09	1.4423E−03	3.1821E−04	7.7387E−06	2.2658E−03	4.6390E−05	1.1711E−02
F21	3.0199E−11	3.0199E−11	3.0199E−11	3.0199E−11	3.0199E−11	2.0152E−08	3.0199E−11	8.1527E−11	4.4205E−06	7.6950E−08	6.2027E−04
F22	3.0199E−11	3.0199E−11	3.0199E−11	3.0199E−11	3.0199E−11	3.0199E−11	3.0199E−11	3.0199E−11	3.0199E−11	3.0199E−11	3.0199E−11
F23	3.3384E−11	3.0199E−11	3.0199E−11	3.0199E−11	3.0199E−11	3.0103E−07	5.4941E−11	3.0199E−11	3.8481E−03	2.1540E−06	**5.7460E−02**
F24	1.4110E−09	3.0199E−11	4.9752E−11	1.0937E−10	6.6955E−11	3.3386E−03	3.8202E−10	8.4848E−09	4.8560E−03	**9.7052E−01**	1.4067E−04
F25	3.0199E−11	1.7769E−10	3.0199E−11	3.0199E−11	3.0199E−11	7.3891E−11	3.0199E−11	1.1023E−08	2.2658E−03	1.2212E−02	**1.8577E−01**
F26	3.6897E−11	3.0199E−11	3.0199E−11	3.0199E−11	3.6897E−11	1.5964E−07	2.6015E−08	2.6806E−04	7.7387E−06	9.8329E−08	2.4994E−03
F27	3.0199E−11	3.0199E−11	3.0199E−11	3.0199E−11	3.0199E−11	3.0199E−11	3.0199E−11	3.0199E−11	3.0199E−11	3.0199E−11	3.0199E−11
F28	3.0199E−11	2.3897E−08	3.0199E−11	3.0199E−11	3.0199E−11	4.5043E−11	3.0199E−11	2.6015E−08	6.2027E−04	1.3017E−03	6.9125E−04
F29	3.0199E−11	3.0199E−11	3.0199E−11	3.0199E−11	3.0199E−11	4.9752E−11	3.0199E−11	3.0199E−11	1.0702E−09	5.4941E−11	1.3111E−08
F30	3.0199E−11	3.0199E−11	3.0199E−11	3.0199E−11	3.0199E−11	3.0199E−11	3.0199E−11	2.6099E−10	3.2555E−07	3.0199E−11	3.6897E−11

Significant values are in [bold].

**Table 11:** P-value of 12 algorithms on CEC 2017 (Dim = 50)

ID	PO	HLOA	LEA	HEOA	NRBO	MELGWO	HPHHO	PPSO	EWOA	SRIME	RIME
F1	3.0199E−11	3.0199E−11	3.0199E−11	3.0199E−11	3.0199E−11	3.0199E−11	3.0199E−11	3.0199E−11	3.0199E−11	3.0199E−11	5.4941E−11
F2	3.0199E−11	3.0199E−11	3.0199E−11	3.0199E−11	3.0199E−11	3.0199E−11	1.2118E−12	3.0199E−11	3.0199E−11	1.6132E−10	3.4742E−10
F3	3.0199E−11	1.0035E−03	1.4643E−10	2.8716E−10	2.6695E−09	3.0199E−11	3.0199E−11	3.0199E−11	1.2362E−03	5.5999E−07	2.6784E−06
F4	3.0199E−11	3.0199E−11	3.0199E−11	3.0199E−11	3.0199E−11	3.0199E−11	3.0199E−11	5.4941E−11	8.1527E−11	2.5306E−04	1.4932E−04
F5	3.0199E−11	3.0199E−11	3.0199E−11	3.0199E−11	3.0199E−11	2.0523E−03	3.0199E−11	4.9752E−11	9.0307E−04	4.8011E−07	3.7704E−04
F6	3.0199E−11	3.0199E−11	3.0199E−11	3.0199E−11	3.0199E−11	3.6897E−11	3.0199E−11	3.0199E−11	8.1527E−11	4.0772E−11	3.6459E−08
F7	3.0199E−11	3.0199E−11	3.0199E−11	3.0199E−11	3.0199E−11	3.6897E−11	3.0199E−11	3.0199E−11	3.6897E−11	1.0702E−09	**3.4783E−01**
F8	3.0199E−11	3.0199E−11	3.0199E−11	3.0199E−11	3.0199E−11	1.0702E−09	3.0199E−11	3.0199E−11	3.1589E−10	5.4941E−11	9.7917E−05
F9	**8.5338E−01**	**7.7312E−01**	3.0199E−11	2.1506E−02	**3.7904E−01**	5.8737E−04	**3.9527E−01**	**6.1452E−02**	**1.9073E−01**	1.1711E−02	3.1821E−04
F10	2.1265E−04	5.2650E−05	6.6955E−11	1.7290E−06	2.5721E−07	**1.0000E+00**	7.9590E−03	**1.0869E−01**	6.6689E−03	**9.3519E−01**	**7.9782E−02**
F11	3.0199E−11	3.0199E−11	3.0199E−11	3.0199E−11	3.0199E−11	3.0199E−11	3.0199E−11	5.4941E−11	3.6897E−11	3.6897E−11	1.4643E−10
F12	3.0199E−11	3.0199E−11	3.0199E−11	3.0199E−11	3.0199E−11	3.3384E−11	3.0199E−11	1.4643E−10	**1.0869E−01**	1.2057E−10	1.6132E−10
F13	3.0199E−11	3.0199E−11	3.0199E−11	3.0199E−11	3.0199E−11	6.5183E−09	3.0199E−11	1.3272E−02	4.1825E−09	2.4386E−09	1.0702E−09
F14	2.8716E−10	7.7387E−06	3.0199E−11	2.9215E−09	9.0632E−08	3.0317E−02	1.2541E−07	**3.8710E−01**	5.8737E−04	**8.4180E−01**	**1.4128E−01**
F15	3.0199E−11	3.0103E−07	3.0199E−11	3.0199E−11	3.0199E−11	1.1937E−06	3.0199E−11	**5.9969E−01**	3.0939E−06	1.9568E−10	2.6099E−10
F16	3.0199E−11	3.0199E−11	3.0199E−11	3.0199E−11	3.0199E−11	9.5332E−07	6.0658E−11	4.9980E−09	5.2640E−04	1.0105E−08	5.8587E−06
F17	3.0199E−11	9.7555E−10	3.0199E−11	4.6159E−10	3.0199E−11	1.3853E−06	3.0811E−08	8.3520E−08	4.0840E−05	5.4620E−06	6.9125E−04
F18	9.7555E−10	**2.0621E−01**	3.0199E−11	3.0199E−11	7.3803E−10	3.7704E−04	1.1077E−06	3.0339E−03	2.5306E−04	1.6955E−02	2.2780E−05
F19	3.0199E−11	3.0199E−11	3.0199E−11	3.0199E−11	3.0199E−11	1.4110E−09	3.0199E−11	9.5207E−04	1.7666E−03	4.9752E−11	4.0772E−11
F20	7.0430E−07	1.7769E−10	3.0199E−11	3.4742E−10	4.6159E−10	**1.1536E−01**	8.5641E−04	5.0922E−08	2.0523E−03	2.3168E−06	1.1143E−03
F21	4.9752E−11	3.0199E−11	3.0199E−11	3.0199E−11	3.0199E−11	2.0023E−06	7.3891E−11	1.2057E−10	7.6588E−05	6.5277E−08	**5.2014E−01**
F22	**7.2827E−01**	**2.4581E−01**	1.4643E−10	**3.8710E−01**	3.1821E−04	3.6709E−03	**3.3285E−01**	**5.1877E−02**	**1.8577E−01**	3.3386E−03	1.5969E−03
F23	3.0199E−11	3.0199E−11	3.0199E−11	3.0199E−11	3.0199E−11	2.6077E−02	3.0199E−11	3.0199E−11	**1.1882E−01**	1.5178E−03	2.5101E−02
F24	1.4110E−09	3.0199E−11	4.9752E−11	4.5043E−11	1.2057E−10	4.0595E−02	2.8716E−10	3.6897E−11	2.6077E−02	**4.0354E−01**	1.5292E−05
F25	3.0199E−11	3.0199E−11	3.0199E−11	3.0199E−11	3.0199E−11	3.0199E−11	3.0199E−11	3.3384E−11	4.9752E−11	1.8500E−08	1.4733E−07
F26	3.6897E−11	3.6897E−11	3.0199E−11	3.0199E−11	4.0772E−11	2.3715E−10	4.5043E−11	4.5043E−11	7.1186E−09	5.4617E−09	8.4848E−09
F27	3.0199E−11	3.0199E−11	3.0199E−11	3.0199E−11	3.0199E−11	3.0199E−11	3.0199E−11	3.0199E−11	3.0199E−11	3.0199E−11	3.0199E−11
F28	3.0199E−11	3.0199E−11	3.0199E−11	3.0199E−11	3.0199E−11	3.0199E−11	3.0199E−11	3.0199E−11	3.0199E−11	1.0702E−09	5.5727E−10
F29	3.0199E−11	3.0199E−11	3.0199E−11	3.0199E−11	3.0199E−11	8.1527E−11	3.0199E−11	5.4941E−11	3.1821E−04	3.4742E−10	5.1857E−07
F30	3.0199E−11	3.0199E−11	3.0199E−11	3.0199E−11	3.0199E−11	3.0199E-11	3.0199E-11	3.0199E-11	3.0199E-11	3.0199E-11	3.0199E-11

Significant values are in [bold].

**Table 12:** P-value of 12 algorithms on CEC 2022 (Dim = 10)

ID	PO	HLOA	LEA	HEOA	NRBO	MELGWO	HPHHO	PPSO	EWOA	SRIME	RIME
F1	3.0199E-11	3.0199E-11	3.0199E-11	3.0199E-11	3.0199E-11	1.5846E-04	3.0199E-11	1.3703E-03	3.0199E-11	4.1997E-10	1.6132E-10
F2	3.0059E-04	8.6844E-03	1.4733E-07	6.0459E-07	3.8053E-07	**1.2967E-01**	4.8560E-03	**2.2257E-01**	**6.5204E-01**	**5.0120E-02**	**2.8378E-01**
F3	3.0199E-11	3.0199E-11	3.0199E-11	3.0199E-11	3.0199E-11	3.0199E-11	3.0199E-11	3.0199E-11	3.0199E-11	3.0199E-11	1.4643E-10
F4	**9.5873E-01**	2.4327E-05	3.6897E-11	1.2541E-07	2.2360E-02	2.5974E-05	**9.8231E-01**	**1.1536E-01**	**1.4532E-01**	**4.3764E-01**	**6.3088E-01**
F5	3.0199E-11	3.0199E-11	3.0199E-11	3.0199E-11	3.0199E-11	7.3891E-11	3.0199E-11	3.0199E-11	3.3384E-11	2.0023E-06	1.0407E-04
F6	2.8716E-10	4.1997E-10	3.0199E-11	7.3891E-11	8.1014E-10	5.9673E-09	1.3111E-08	2.7548E-03	7.0881E-08	1.5465E-09	2.0338E-09
F7	3.0199E-11	3.0199E-11	3.0199E-11	3.0199E-11	3.0199E-11	1.2057E-10	1.6947E-09	6.7220E-10	1.1023E-08	4.5043E-11	2.6784E-06
F8	3.0199E-11	3.0199E-11	3.0199E-11	3.0199E-11	3.0199E-11	3.9648E-08	3.0199E-11	1.0937E-10	2.8389E-04	5.0723E-10	3.7782E-02
F9	3.0199E-11	3.0199E-11	3.0199E-11	3.0199E-11	3.0199E-11	3.0199E-11	3.0199E-11	3.0199E-11	3.0199E-11	3.0199E-11	3.0199E-11
F10	1.8575E-03	2.1540E-06	2.1327E-05	4.5043E-11	2.9590E-05	4.8560E-03	4.0330E-03	1.1058E-04	7.9590E-03	1.4932E-04	**1.1199E-01**
F11	1.7290E-06	1.6980E-08	1.4110E-09	1.4918E-06	2.0338E-09	4.6371E-03	1.6351E-05	**6.7869E-02**	1.9883E-02	6.5261E-07	4.7445E-06
F12	5.5727E-10	1.7769E-10	3.4742E-10	4.1997E-10	5.0723E-10	5.0723E-10	5.0723E-10	3.8202E-10	5.5727E-10	5.0723E-10	5.5727E-10

Significant values are in [bold].

**Table 13:** P-value of 12 algorithms on CEC 2022 (Dim = 20)

ID	PO	HLOA	LEA	HEOA	NRBO	MELGWO	HPHHO	PPSO	EWOA	SRIME	RIME
F1	**1.7613E-01**	7.9590E-03	3.0199E-11	6.1210E-10	1.9883E-02	3.0059E-04	1.0763E-02	4.8011E-07	1.7294E-07	3.3384E-11	6.0658E-11
F2	3.0199E-11	2.0023E-06	3.0199E-11	3.0199E-11	3.0199E-11	3.6459E-08	3.3384E-11	1.7479E-05	7.6973E-04	1.0188E-05	3.5012E-03
F3	3.0199E-11	3.0199E-11	3.0199E-11	3.0199E-11	3.0199E-11	3.0199E-11	3.0199E-11	3.0199E-11	7.3891E-11	3.3384E-11	1.1023E-08
F4	6.7362E-06	1.3367E-05	3.0199E-11	1.6132E-10	2.1544E-10	4.4592E-04	2.1566E-03	4.8413E-02	8.3146E-03	**3.2553E-01**	1.2212E-02
F5	3.8053E-07	1.1567E-07	3.0199E-11	1.3111E-08	7.0430E-07	4.3531E-05	1.7294E-07	1.0277E-06	7.7387E-06	7.1988E-05	6.3772E-03
F6	3.6897E-11	2.2780E-05	3.0199E-11	3.0199E-11	3.0199E-11	7.9590E-03	3.0199E-11	8.3146E-03	3.6709E-03	3.9648E-08	9.7555E-10
F7	1.4294E-08	4.0772E-11	4.0772E-11	2.3715E-10	1.5581E-08	8.6634E-05	1.3367E-05	2.0023E-06	2.8129E-02	1.8608E-06	**1.2597E-01**
F8	2.0058E-04	1.4110E-09	5.4941E-11	2.1506E-02	1.9963E-05	6.6689E-03	4.5146E-02	2.0523E-03	**2.0095E-01**	9.5207E-04	**2.5188E-01**
F9	3.0199E-11	3.0199E-11	3.0199E-11	3.0199E-11	3.0199E-11	3.0199E-11	3.0199E-11	3.0199E-11	3.0199E-11	3.0199E-11	3.0199E-11
F10	3.3384E-11	3.0199E-11	3.0199E-11	3.0199E-11	3.0199E-11	9.9186E-11	9.9186E-11	3.0199E-11	4.1997E-10	4.5043E-11	4.4440E-07
F11	3.0199E-11	2.0152E-08	3.0199E-11	3.0199E-11	3.0199E-11	7.6588E-05	3.0199E-11	1.1228E-02	**2.0095E-01**	7.6588E-05	1.0907E-05
F12	3.0199E-11	3.0199E-11	3.0199E-11	3.0199E-11	3.0199E-11	3.0199E-11	3.0199E-11	3.0199E-11	3.0199E-11	3.0199E-11	3.0199E-11

Significant values are in [bold].

**Table 14:** Wilcoxon rank sum test statistical results

MIRIME VS	CEC2017 (Dim = 10)	CEC2017 (Dim = 30)	CEC2017 (Dim = 50)	CEC2022 (Dim = 10)	CEC2022 (Dim = 20)
PO	27/0/3	29/0/1	28/0/2	11/0/1	11/0/1
HLOA	28/0/2	29/0/1	27/0/3	12/0/0	12/0/0
LEA	30/0/0	30/0/0	29/0/1	12/0/0	12/0/0
HEOA	28/0/2	29/0/1	29/0/1	12/0/0	12/0/0
NRBO	29/0/1	30/0/0	29/0/1	12/0/0	12/0/0
MELGWO	22/0/8	30/0/0	28/0/2	11/0/1	12/0/0
HPHHO	28/0/2	30/0/0	28/0/2	11/0/1	12/0/0
PPSO	24/0/6	27/0/3	25/0/5	9/0/3	12/0/0
EWOA	22/0/8	28/0/2	26/0/4	10/0/2	10/0/2
SRIME	28/0/2	28/0/2	27/0/3	10/0/2	11/0/1
RIME	21/0/9	23/0/7	26/0/4	9/0/3	10/0/2
Overall(+ / = /-)	**287**/0/43	**313**/0/17	**302**/0/28	**119**/0/13	**126**/0/6

Significant values are in [bold].

#### Friedman rank test

We utilized the nonparametric Friedman mean rank test^63^ to rank the numerical optimization performance of the MIRIME algorithm and other optimizers on the CEC 2017 and CEC 2022 test suites, and the detailed results are reported in Table 15. MIRIME consistently ranks first in the ranking, that highlights that our proposed optimizer significantly outperforms other competing algorithms on the selected test suites.

**Table 15:** The Friedman mean rank test was performed on the considered test suite

Suites	CEC 2017						CEC 2022			
Dimensions	10		30		50		10		20	
Algorithms	Ave.Rank	Overall Rank	Ave. Rank	Overall Rank	Ave. Rank	Overall Rank	Ave. Rank	Overall Rank	Ave. Rank	Overall Rank
PO	7.73	8	8.40	8	8.40	9	7.75	8	7.75	8
HLOA	9.30	10	8.63	9	8.20	8	9.67	10	8.92	9
LEA	11.50	12	11.63	12	11.73	12	11.58	12	11.67	12
HEOA	10.40	11	10.43	11	10.27	11	10.42	11	10.17	11
NRBO	8.27	9	9.40	10	9.70	10	8.75	9	9.25	10
MELGWO	4.47	5	4.77	5	4.53	5	3.75	4	4.50	4
HPHHO	6.93	7	7.00	7	6.93	7	6.42	7	6.67	7
PPSO	5.50	6	5.23	6	5.10	6	5.92	6	5.75	6
EWOA	3.77	2	3.77	3	3.87	3	3.58	2	3.42	3
SRIME	4.20	4	4.40	4	4.03	4	5.17	5	5.00	5
RIME	3.90	3	2.87	2	3.00	2	3.67	3	3.00	2
MIRIME	**2.03**	**1**	**1.47**	**1**	**2.23**	**1**	**1.33**	**1**	**1.92**	**1**

Significant values are in [bold].

### Effectiveness analysis of the improvement

**Figure 14:**
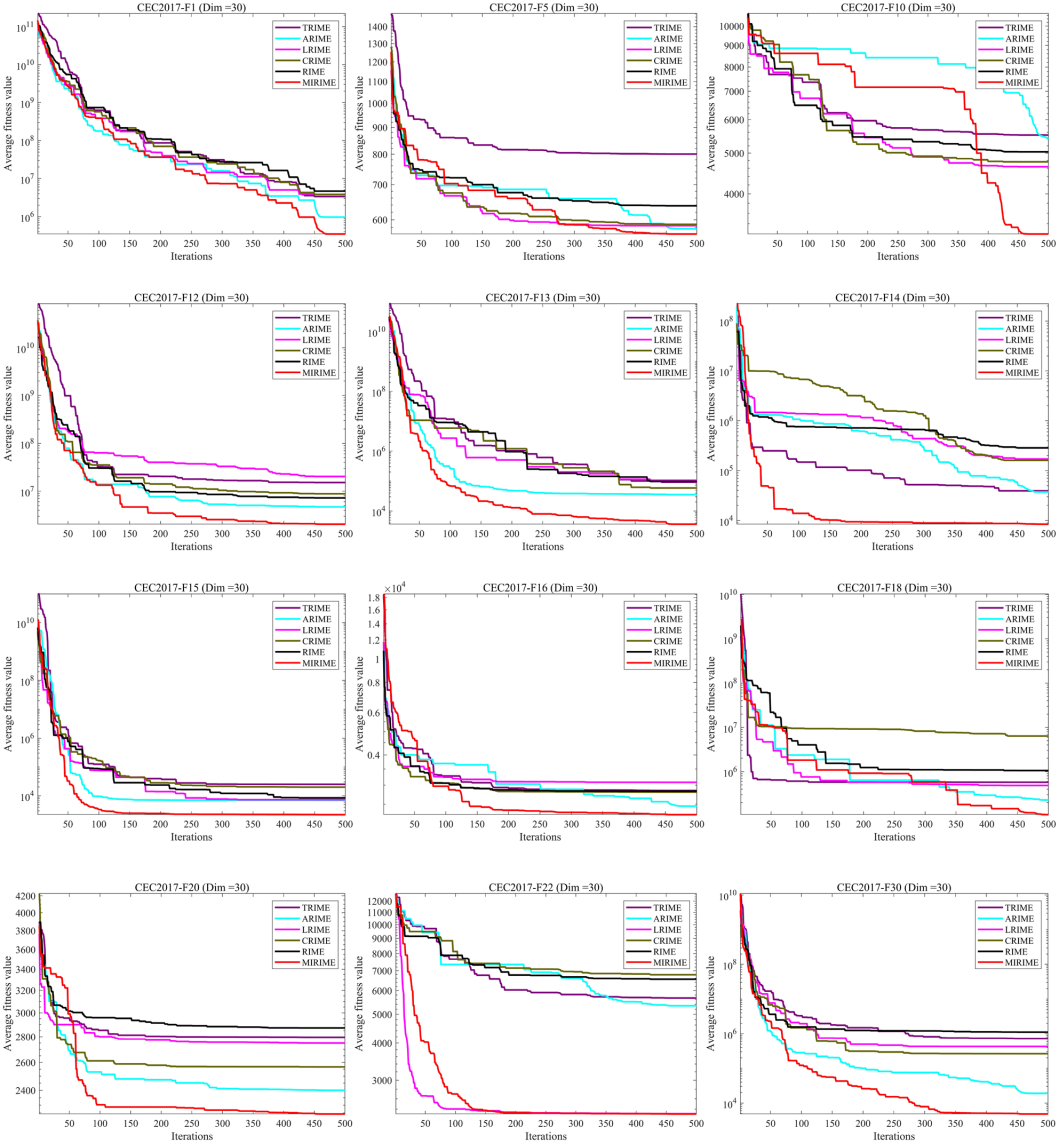
Comparison of different improvement strategies.

In this section, we examine the effectiveness of the proposed improvement strategies on the CEC2017 (Dim = 30) test suites, which include TRIME, ARIME, LRIME, CRIME. The experimental results, displayed in Fig. 14, clearly demonstrate the positive effects of these improvement strategies in enhancing the performance of the original RIME. However, when applied to certain test functions, a single improvement strategy often fails to yield ideal results. In contrast, MIRIME, which incorporates these four strategies, exhibits significant advantages in avoiding local optima and premature convergence, and markedly outperforms RIME. It is evident that the development of the MIRIME algorithm is crucial for enhancing algorithmic performance and represents an indispensable direction for progress.

## MIRIME algorithm for 3D path planning of unmanned vessel

### Establishment of mathematical model

#### Voyage distance cost

In USV path planning, the cost associated with the navigation distance is mainly related to the fuel consumption during the USV voyage^64^. Assuming that the USV achieves and maintains a constant speed during its mission, fuel consumption is proportional to the total distance traveled by the USV. The mathematical expression is as follows:

20 $$\begin{array}{c}{f}_{range}=\frac{\varepsilon }{{\mathcal{Q}}_{r}}\sum_{i=1}^{n} {L}_{i}\end{array}$$

where $\varepsilon$ is unit distance of fuel consumption, ${\mathcal{Q}}_{r}$ denotes unmanned ship carrying fuel, ${L}_{i}$ represents the *ith* segment length.

#### Navigation altitude cost

With the elevation of the unmanned vessel, the probability of low temperature impact also increases. In order to effectively control the risks, made the highest ${h}_{max}$ and the lowest ${h}_{min}$ navigation altitude restriction. In setting the unmanned ship sailing elevation of ${h}_{i}$, corresponding navigation above cost function can be expressed as:

21 $$f_{{H_{i} }} = f_{{H_{i} }} = \left\{ {\begin{array}{*{20}c} {\frac{{h_{i} - h_{min} }}{{h_{max} - h_{min} }}, } & {h_{min} < h_{i} < h_{max} } \\ {\infty ,} & {others} \\ \end{array} } \right.$$

22 $$\begin{array}{c}{f}_{ \, {\text{altitude}} \, }=\frac{1}{n}\sum_{i=1}^{n} {f}_{{h}_{i}}\end{array}$$

#### Navigation risk cost

The route of an unmanned ship may traverse a variety of risky areas, including rough terrain and extreme weather. These risk costs are evaluated based on the distance of the unmanned vessel from these risk sources during the voyage. If a particular navigation section ${L}_{i}$ is divided a small section $m$, and in the near a risk point $k$, you will need to calculate the flight due to close to the risk point $k$ risk cost. Flight segment ${L}_{i}$ due to close to the risk point $k$ risk cost function can be expressed as Eq. (23).

23 $$\begin{array}{c}{f}_{{n}_{i,k}}=\frac{1}{m}\left({P}_{k}\left({d}_{k,1}\right)+{P}_{k}\left({d}_{k,2}\right)+\cdots +{P}_{k}\left({d}_{k,m}\right)\right)\end{array}$$

where ${P}_{k}$ represents the probability that the unmanned ship is destroyed by the *k*^*th*^ threat point, ${d}_{k,m}$ denotes the distance from the *k*^*th*^ threat point to the *m*^*th*^ sub-section in the road ${L}_{i}$. The total threat cost generated by path planning of unmanned vessel can be expressed as follows.

24 $$\begin{array}{c}{f}_{risk}=\frac{1}{n}\frac{1}{k}\sum_{i=1}^{n} \sum_{i}^{k} {f}_{{n}_{i},k}\end{array}$$

#### Total cost calculation of unmanned vessel path planning

The path planning of unmanned ships involves three main cost factors: navigation distance, navigation altitude, and navigation risk. These cost factors are assigned weight coefficients (ω1, ω2, ω3). Based on these, the objective function of route planning for unmanned vessels is transformed into a weighted sum of these cost elements. This formula is designed to balance various factors in order to find the best route. The objective function of unmanned ship route planning is defined as follows:

25 $$\begin{array}{c}f={\omega }_{1}{f}_{range}+{\omega }_{2}{f}_{\text{altitude}}+{\omega }_{3}{f}_{\text{risk}}\end{array}$$

### Simulation experiment analysis

**Figure 15:**
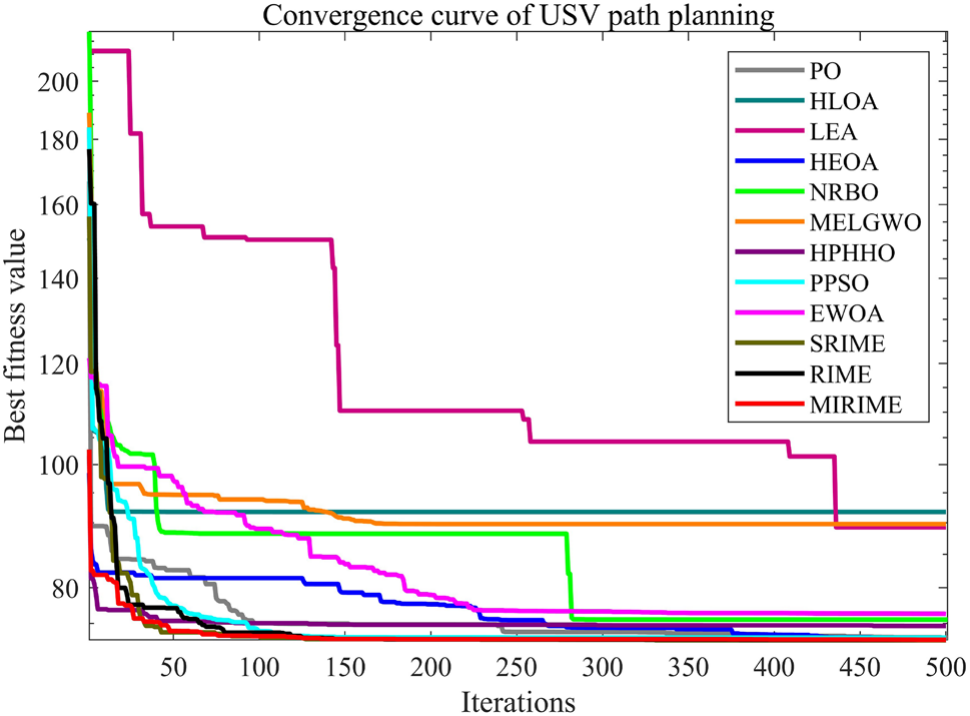
Comparison of convergence curves of different competitors.

**Figure 16:**
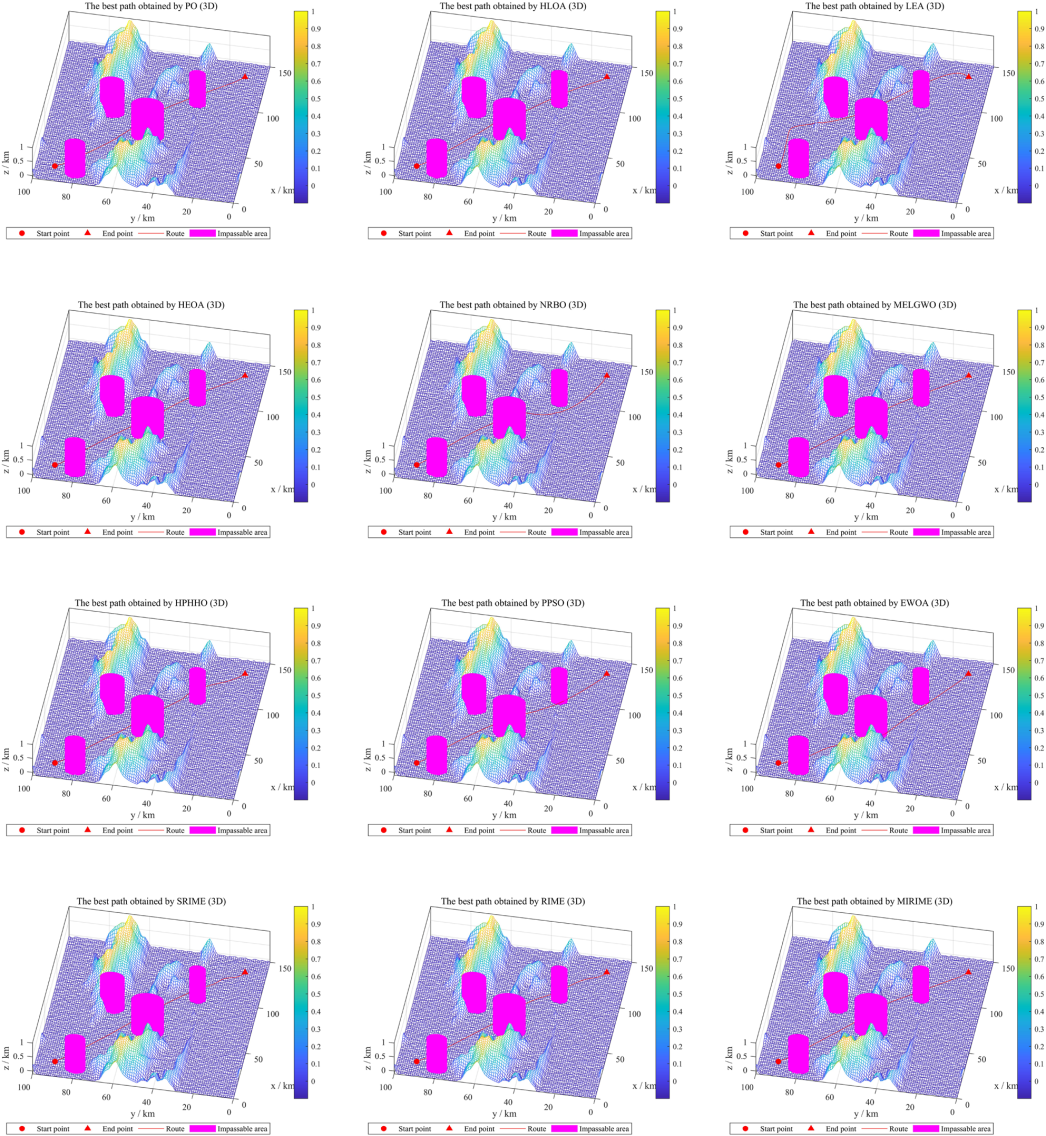
Optimal path obtained by different algorithms (3D).

**Figure 17:**
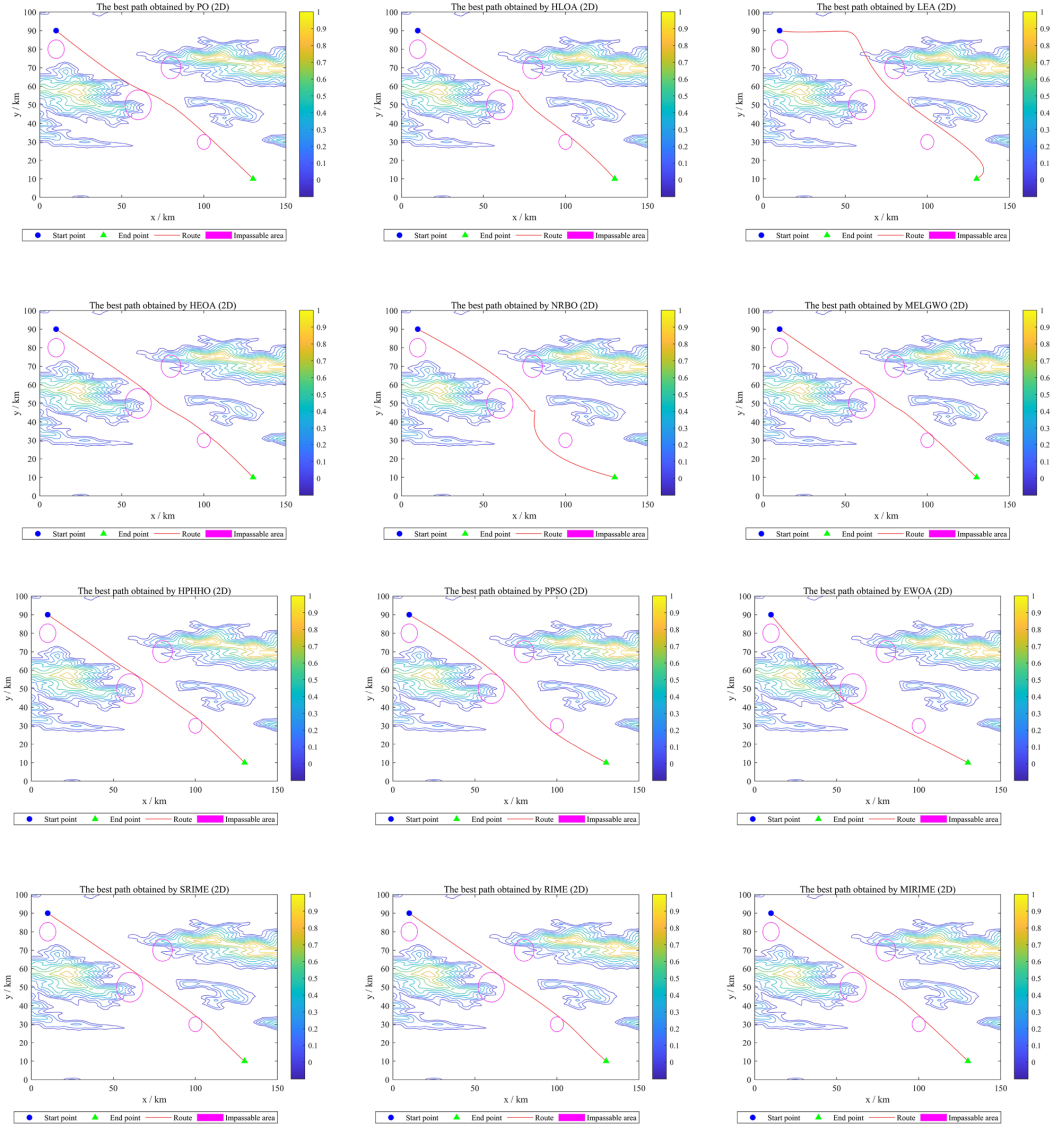
Optimal path obtained by different algorithms (2D).

In this section, we apply the MIRIME algorithm to solve the unmanned vessel path planning problem. We record the Best solution (Best), the median value (Median), the Worst solution (Worst), the average cost (Ave), and the standard deviation (Std), with the best results highlighted in bold. It is evident from Table 16 that our algorithm performs well in terms of both cost and stability. Figure 15 depicts the convergence process, illustrating MIRIME's faster convergence rate. Additionally, Figs. 16 and 17 show the optimal 3D and 2D paths obtained by different algorithms, respectively. It is apparent that our proposed algorithm generates shorter and smoother paths, further validating the effectiveness of MIRIME in solving practical problems.

**Table 16:** Experimental results of competitors in USV 3D trajectory planning

Algorithms	Best	Median	Worst	Ave	Std	Friedman ranking
PO	72.98197	73.39766	75.33018	73.58412	0.58877	5
HLOA	73.64427	82.90435	88.97828	82.83909	3.52121	7
LEA	75.95002	96.05761	**109.16357**	94.26089	8.66003	10
HEOA	72.82092	73.46824	75.46086	73.60773	0.58494	12
NRBO	73.39278	80.12478	92.30395	81.07048	4.25704	8
MELGWO	72.74539	**72.81782**	73.29205	72.92435	0.17411	6
HPHHO	72.76649	72.82628	73.09887	72.86523	0.61011	11
PPSO	72.72655	72.88907	73.74893	72.96328	0.22870	3
EWOA	72.78995	76.19436	83.50781	75.88499	2.27531	9
SRIME	72.73468	72.86882	73.39545	72.94407	0.17442	4
RIME	72.72936	72.82440	73.35683	72.90080	0.16560	2
MIRIME	**71.03586**	72.88080	73.13354	**72.65595**	**0.08869**	**1**

Significant values are in [bold].

## Summary and outlook

In this paper, we propose a multi-strategy improved RIME algorithm (MIRIME) to address the issue of RIME algorithm's susceptibility to falling into local optima. In the initial stage, Tent mapping is introduced to initialize the population (TRIME), ensuring a more even distribution of individuals across the search space, thereby facilitating global search. During the soft rime search (exploration) stage, we introduce an adaptive update rule based on the leader and dynamic centroid (ARIME). The leader guides the algorithm towards the currently considered optimal direction, while the dynamic centroid serves as a reference point reflecting group concentration, aiding individuals in maintaining collaboration with the swarm during exploration. To address the issue of decreased population diversity leading to low convergence accuracy in later iterations of RIME, we introduce the lens imaging opposition-based learning mechanism to improve optimization ability (LRIME). Additionally, the centroid boundary control mechanism effectively enhances search focus and efficiency by limiting individual search boundaries and using the population centroid as a guide point (CRIME). This strategy not only prevents exploration in invalid regions, reducing resource waste, but also ensures group concentration in high-potential areas, promoting the discovery of high-quality solutions. We evaluate the performance of MIRIME using 30 test functions from CEC2017 and 12 test functions from CEC2022, demonstrating its effectiveness across different dimensions of test functions. Through Wilcoxon and Friedman test statistical analysis, we confirm MIRIME's significant superiority over competitors. To validate MIRIME's effectiveness in solving practical problems, we apply it to the three-dimensional path planning problem of unmanned surface vehicles. In the future, the MIRIME improvement strategy will be applied to other algorithms, and consider developing a multi-purpose version. In addition, MIRIME is also planned to be applied to practical problems such as document classification, data mining, image segmentation, data clustering, analog circuit fault diagnosis, wireless sensor networks, and three-dimensional reconstruction.

## Data Availability

All data generated or analysis during this study are included in this published article.
